# Implementation of diabetes prevention programs into clinical practice and community settings: a systematic search and review

**DOI:** 10.1186/s43058-025-00757-2

**Published:** 2025-07-09

**Authors:** Tineke E. Dineen, Corliss Bean, Azar Bohlouli, Sarah L. Percival, Mathew Vis-Dunbar, Mary E. Jung

**Affiliations:** 1 School of Health and Exercise Sciences, University of British Columbia, Okanagan Campus, 1238 Discovery Avenue, Kelowna, BC V1V 1V7 Canada; 2 Department of Recreation and Leisure Studies, Brock University, St Catharines, ON Canada; 3 UBC Library, University of British Columbia, Okanagan Campus, Kelowna, BC Canada

**Keywords:** Diabetes prevention, Prediabetic state, Implementation science, Implementation strategies, Implementation outcomes, Implementation determinants

## Abstract

**Background:**

Greater understanding of how evidence-based programs have been implemented in clinical practice and community settings is needed. Implementation science can help understand how to best implement programs, however, the fast-developing field is hindered by inconsistent terminology and reporting. To increase transparency and improve implementation science, standardized tools have been created. The aim of this systematic search and review was to identify implementation strategies, outcomes and determinants using standardized tools when diabetes prevention programs were implemented within a clinical practice and community setting.

**Methods:**

A comprehensive peer-reviewed search strategy was used to identify relevant articles. Relevant studies were retrieved from four electronic databases and specific inclusion and exclusion criteria were applied. Implementation strategies, outcomes, determinants, and theoretical frameworks were extracted from all included articles using two standardized tools (the refined compilation of implementation strategies and the minimum dataset of implementation determinants and outcomes). Data from the extraction tool were summarized using a narrative approach. Frequency of reported implementation strategies, outcomes, determinants, and theoretical frameworks are presented.

**Results:**

Retrospective researcher extraction resulted in the representation of 69 of the 73 implementation strategies. An average of 13.8 strategies (± 9.1) were reported, programs ranged from zero to 41 strategies. The most common reported strategies included: *conduct educational meetings, build a coalition,* and *promote adaptability*. Individual implementation determinants and outcomes were not extracted due to the difficulty applying standardized definitions to the dataset and the limited implementation data. Most studies (75%) lacked a theoretical framework.

**Discussion:**

Significant gaps exist in reporting implementation strategies, providing sufficient detail on how implementation projects are implemented, and researching implementation variables within diabetes prevention programs. Large implementation projects contained more implementation strategies and variables than small projects. The use of standardized tools for the extraction of implementation strategies, outcomes, and determinants was difficult due to insufficient detail provided in existing literature on how programs have been implemented and ambiguity in standardized tool definitions. To build the field of implementation science, researchers must report sufficient detail on how programs have been implemented and research implementation variables.

**Trial registration:**

This systematic search and review was registered on Open Science Frameworks and can be accessed with this link: https://osf.io/cbzja.

**Supplementary Information:**

The online version contains supplementary material available at 10.1186/s43058-025-00757-2.

Contributions to the literature

- Findings reiterate that implementation strategies are rarely reported and help fill this gap by retrospectively identifying implementation strategies from published diabetes prevention programs.
- Available standardized tools need further development to reduce ambiguity, be more comprehensive, and facilitate use.
- Findings highlight commonly used implementation strategies and highlight areas for future research.
- Only 20% of programs reported implementation outcomes, mostly at the program level. Findings highlight a large gap of research failing to investigate implementation outcomes at the implementation strategy level.
- Only 20% of programs reported implementation determinants, yet implementation determinants were investigated from both program and implementation strategy levels.

## Background

Type two diabetes (T2D) is among the fastest growing global health condition in the world. Diet and exercise are two modifiable risk factors that have repeatedly been shown to have a potent effect on reducing progression from prediabetes to T2D [1–4], with long-lasting effects [1, 5]. Diabetes prevention programs (DPP), of which the majority target diet and exercise modifications, have been translated into clinical practice and community settings demonstrating effectiveness and cost-effectiveness [6, 7]. This paper used the acronym DPP to refer to any program that targets diabetes prevention and is not to be confused with the United States DPP, one very specific program. Numerous DPPs exist that use a variety of lower cost strategies for clinical and community settings (e.g., group-based formats, using lay community members to deliver a DPP over more costly health care providers, shorter program duration, less frequency of sessions) and DPPs have been implemented across the world in hospital, work, community, and school settings [8]. Despite the known benefits of DPPs and the increasing prevalence of prediabetes, widespread implementation of DPPs is needed.

### Implementation strategies

A challenge exists in knowing how to effectively move evidence-based initiatives to resource-constrained and diverse settings. Implementation science studies the methods used to promote uptake of evidence into practice [9]. In the past decade there has been a dramatic increase in implementation science frameworks, methodology, and tools. One such development is the Expert Recommendations for Implementing Change (ERIC) compilation of 73 implementation strategies that standardizes a language for reporting implementation strategies [10]. Implementation strategies are the specific methods, actions, or techniques to facilitate the movement of evidence-based initiatives into clinical practice and community settings [11]. Implementation strategies include *distributing educational materials, developing an implementation blueprint, implementation team meetings*, *developing quality monitoring systems* etc*.* [10]. To our knowledge, no review to date has extracted implementation strategies from DPPs. Identifying implementation strategies from prior research will make a novel contribution to understanding how programs are being implemented, identify gaps in the literature, develop an evidence base, and make recommendations for effectively implementing programs.

### Implementation outcomes and determinants

Evidence-based programs must be matched with evidence-based implementation [12]. In a resource and time-constrained world, implementation is often overlooked in favour of program effectiveness outcomes or program recipient feedback. A type III error may result when implementation is not examined: when a program considered ineffective may have been effective, had appropriate implementation occurred [13]. Investigating implementation variables (i.e., outcomes and determinants) are essential to explain program effectiveness, especially within clinical practice and community settings that aim to increase generalizability of results [14, 15]. To better understand implementation, research in clinical practice and community settings should collect implementation data from multiple perspectives at varying levels (e.g., delivery team, sites, organization) [16–18]. Data collection and analysis that extends beyond traditional program effectiveness or program recipient feedback is needed.

To guide researchers on which implementation variables to examine, McKay and colleagues created a minimum dataset for implementation evaluations for physical activity and nutrition programs (hereafter referred to as implementation dataset) [17]. The implementation dataset was created using Delphi consensus methodology with an international group of implementation scientists [17]. The work identifies implementation determinants (factors that affect implementation) and outcomes (results from the implementation) and specifies that implementation determinants and outcomes must be researched from both delivery of the intervention (program) and delivery of implementation strategy (e.g., ERIC strategies) levels [17]. Measuring the same variable at two levels enables a detailed understanding of how an implementation initiative was executed. For example, fidelity, designated as an implementation outcome, should be researched at both the program level (i.e., extent to which the intervention was delivered as planned by the delivery team) and the implementation strategy level (i.e., extent to which the implementation strategies are delivered as planned by the support team) [17]. Measuring variables at both levels is a valuable practice within implementation evaluations, yet the extent to which variables are reported at these levels is unknown.

To our knowledge, no previous review has examined to what extent implementation determinants and outcomes have been reported at the program and implementation level. One prior review examined a narrow range of implementation outcomes and did not examine implementation determinants nor did the review report the level the implementation outcomes were measured at (program or implementation level) [19]. Another review examined implementation outcomes at both the individual and organizational levels but did not examine implementation determinants [20]. The review focused only on DPPs in the United States (U.S.) that were based on the U.S. DPP [20], resulting in a narrow scope and worldwide applicability. For a comprehensive understanding of the current state of DPP implementation literature worldwide, a new review that examines both implementation outcomes and determinants at program and implementation strategy levels using a standardized tool is needed.

Overall, this review provides a holistic synthesis of how DPPs have been implemented and researched. The aim of this exploratory review is to describe the state of the literature and provide recommendations for implementation research in DPPs. This review had two co-primary research questions, ‘(1) What implementation strategies, as defined by the ERIC compilation, have been used when implementing a diet and/or exercise DPP into clinical practice and community settings?’ and ‘(2) What implementation determinants and outcome variables, as defined by McKay and colleagues, have been reported when implementing a diet and/or exercise DPP into clinical practice and community settings, and at what level(s)?’. In addition, this review had one secondary research question, ‘What implementation theories, models or frameworks have been used when implementing a diet and/or exercise DPP into a clinical practice and community settings, and for what purpose?’.

## Methods

In consultation with a research librarian we selected a systematic search and review methodology as defined by Grant and Booth [21]. This review type is characterized by a comprehensive search strategy that attempts to identify what is known on a specific topic, provide recommendations for practice, and is suitable when quality assessment of included studies is not in scope [21]. This review type combines strengths from both scoping and systematic review methodologies. Like a scoping review, this review type is well-suited to synthesize evidence for a broad research question, extracting data from a variety of study designs to understand prevalence of research on a topic [21]. However, this review needed a comprehensive and specific search strategy, that was reflective of a systematic review as opposed to a scoping review [21]. To increase methodological rigour, the Preferred Reporting Items for Systematic Reviews and Meta-Analysis (PRISMA) guidelines were used and modified as necessary to fit this specific review type [22] (see Additional file 1). The review protocol was registered on Open Science Frameworks (see https://osf.io/cbzja) [23].

### Search strategy

A search strategy was developed in collaboration with a research librarian and reviewed by a second research librarian [24]. The final search strategy targeted key terms related to diabetes prevention, programs, and implementation, and was tailored to four electronic databases used for the search (Medline, Embase, Web of Science and Google Scholar) and run on Oct 19, 2021. Reference lists of relevant published reviews and all included papers were examined for additional relevant literature. Refer to the protocol for full search strategy details [23].

### Eligibility criteria

Specific inclusion and exclusion criteria were applied based on the population, intervention, comparison, outcomes, and study type (PICOS; see protocol [23]). This review examined the implementation of DPPs, as such the population of interest are the programs that are characterized by the inclusion of individuals at risk of T2D. Thus, published articles on DPPs that identify individuals as at risk of developing T2D by one of the following methods were included: (1) by a blood test including either a HbA1c, fasting blood glucose, or oral glucose tolerance test indicating impaired fasting glucose or impaired glucose tolerance as defined by the study, (2) a standardised risk assessment test that identified them at increased risk for T2D (e.g., CANRISK, FINDRISK, AUSDRISK, Indian Diabetes Risk Score), or (3) a previous gestational diabetes diagnosis. The program must be facilitated by non-research staff (i.e., community staff conduct the sessions with program recipients), in clinical practice or community, in-person, settings. Implementation of in-person programs differ from programs that use technology (e.g., cost, scalability, human resources, uptake) [25, 26]. Thus, the review focused on a homogenous sample of in-person programs. All studies that detail a program which targets diet and/or exercise modifications to reduce the risk for T2D in at-risk individuals 18 + years old regardless of date, design, or duration were included. If a program included additional targets (e.g., mindfulness), these programs were included. Awareness raising or educational campaigns (e.g., workshops, webinars) were excluded. Exclusion criteria included programs for individuals with a T2D diagnosis, non-human subjects, research staff facilitating the program directly to program recipients, use of technology to deliver the program (e.g., telehealth, digital programs, mobile health programs) and in a laboratory, university, or experimental setting. Due to time and resource constraints, non-English languages articles were excluded. No exclusions were made based on year of publication.

### Study selection

Identified articles were imported into Covidence [27] for data management. After removing duplicates, two reviewers independently screened all titles/abstracts, and full texts to identify potentially eligible studies. To reduce bias and errors, both reviewers met after reviewing 20 title/abstracts and 5 full text manuscripts to improve accuracy during the screening process. At each stage (title/abstract, and full text review) discrepancies were discussed among the two reviewers until consensus was reached. Interrater reliability statistics (% agreement) were assessed at each stage (title/abstract [89%] and full text review [77%]).

### Study quality assessment

Consistent with the review type, a risk of bias assessment was not conducted [21].

### Data extraction

Each publication was extracted individually. Any program referenced within multiple publications were combined at the data analysis stage. Two reviewers independently extracted data from included studies. A customized data extraction tool was created and piloted on the first five publications to improve accuracy and precision during the extraction process. Discrepancies were discussed among the two reviewers until consensus was reached. General study details (program name, country, study design, target behaviour, program setting, number of sites, program type, program duration, number of sessions) were extracted.

#### Implementation strategies

Implementation strategies were extracted using names and definitions from the compilation of implementation strategies (ERIC) [10]. To strengthen coding reliability, a codebook was created to assist consistent application of definitions (Additional file 2). To further describe the results, strategies were grouped according to thematic clusters and grouped into four quadrants according to important and feasibility ratings identified in previous work by authors of the ERIC compilation [28].

#### Implementation outcomes and determinants

An attempt was made to extract implementation determinants and outcomes according to definitions from the implementation dataset (Table 2 of McKay et al.) [17]. Efforts were limited by the small number of studies that reported implementation or implementation strategy level factors and the difficulty applying definitions [17]. For example, it was difficult to confidently assign generic interview data (i.e., facilitators and barriers to implementation) to specific variables based on the implementation dataset definitions (e.g., feasibility, satisfaction, adaptability). Therefore, a broader level of data extraction had to be used. Using the implementation dataset [17] as a guide, the number of studies that reported data at the implementation determinant or outcome levels were extracted. Next, each study was assessed for whether data were reported at the level of the program or implementation strategy. Conclusions could still be made based on the broader level of data extraction.

Additional descriptive details were extracted including whether the following were reported: site or staff descriptive data, interview or focus group data, program effectiveness data, implementation data and finally, the level of research. Level of research was determined by examining the perspective of the target data collection as the participant, facilitator, managerial, site, organization, community, cost, or reflections on implementation. For example, if staff were interviewed, but all interview questions reflected staff perspectives on program recipients, participant level was selected. If interviews with staff reflected the opinions of staff on program implementation, then staff level was selected. Multiple levels could be selected for one publication.

#### Implementation theories, models, and frameworks

Any theory, model, or framework explicitly referenced in an included study as being used for implementation research purposes were extracted and categorized according to the purpose (study design, data collection, or analysis). In addition, each theory, model, or framework was further categorized into on of five categories (process, determinant, classic, implementation, evaluation) of implementation theories, models and frameworks using Nilsen and colleagues as a guide [29].

### Data analysis

Data was descriptively analyzed to describe the presence of implementation strategies, implementation outcomes and implementation determinants.

## Results

**Fig. 1:**
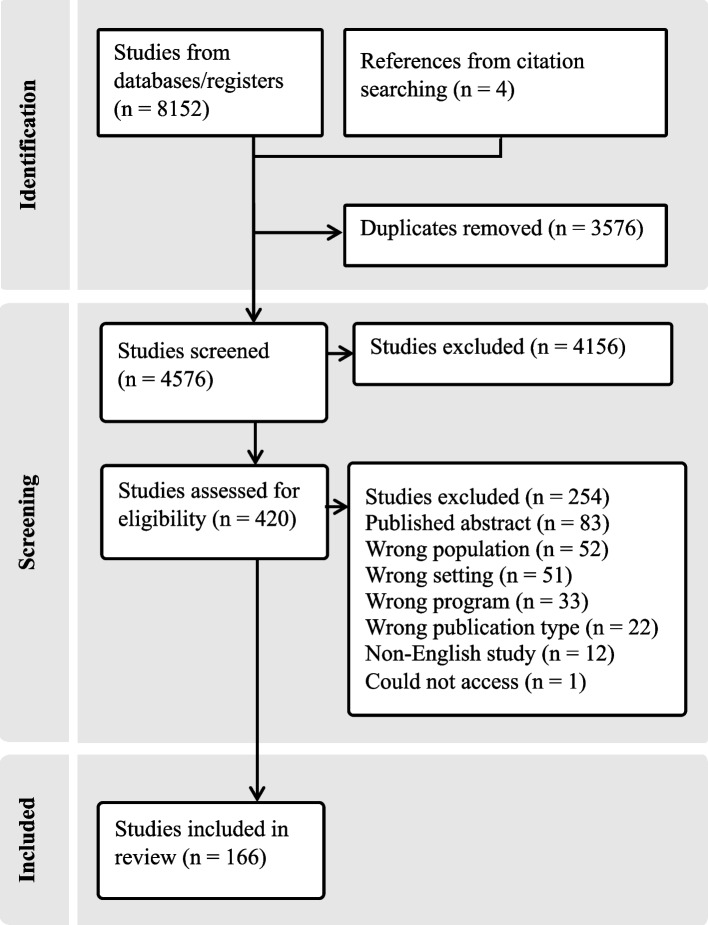
PRISMA flowchart

The search identified 8152 records and 3576 duplicates were removed. A total of 4576 unique studies were screened for eligibility by title and abstract and 420 studies were screened for eligibility by full-text (see Fig. 1 for full PRISMA flowchart and reasons for study exclusion).

### Study (*N* = 166) characteristics

Ultimately, 166 studies were included. Included studies were descriptive (*N* = 36, 22%), cohort (*N* = 28, 17%), pilot/feasibility (*N* = 22, 13%), randomized trials (*N* = 21, 13%), evaluation (N = 19, 11%), design (*N* = 13, 8%), reflection (*N* = 13, 8%), hybrid-implementation-effectiveness (*N* = 4, 2%), cost-effectiveness (*N* = 4, 2%), quasi-experimental (*N* = 3, 2%), case study (*N* = 2, 1%) or cross-sectional (*N* = 1, 1%).

### Program (*n* = 94) characteristics

The 166 included studies represented 94 programs (see Table 1 for program characteristics). Overall, programs were conducted in the U.S. (*n* = 49, 52%), United Kingdom (*n* = 10, 11%), the Netherlands (*n* = 6, 6%), Australia (*n* = 4, 4%), Spain (*n* = 4, 4%), Canada (*n* = 4, 4%), Finland (*n* = 2, 2%), New Zealand (*n* = 2, 2%), Thailand (*n* = 2, 2%), and other countries (Austria, Brazil, China, Denmark, Germany, Greece, Israel, India, Poland, Saudi Arabia, Uganda/South Africa/Sweden, [ *n* = 1, 1%]). Programs were set in primary care (*n* = 46, 49%), community (e.g., church, fitness center, library; *n* = 32, 34%), a combination (*n* = 11, 12%), or workplace settings (*n* = 4, 4%). A minority did not describe the setting (*n* = 1, 1%). Most programs targeted diet and physical activity (*n* = 89, 94%) with one program targeting only dietary behaviours (1%) and five programs targeting diet, physical activity, and an additional behaviour (e.g., alcohol, smoking, sleep, stress; 5%). Program duration was for a year (*n* = 47, 50%), less than a year (*n* = 37, 39%), more than a year (*n* = 5, 5%), or not described (*n* = 5, 5%).

**Table 1:** Program characteristics

Program Name	Citations	Country	Target behaviour	Program setting	# of sites	Program type	Program duration	# of sessions
Project Reaching Immigrants through Community Empowerment (RICE)	[30, 31]	United States	Other: Diet, PA, stress and family access to healthcare	Community	1	Combination of group and individual	Less than a year	16
National Health Service Diabetes Prevention Program	[32]	United Kingdom	Diet and physical activity	Primary care	9	Group	Less than a year	13
Diabetes Prevention in Chinese Women at Risk for Diabetes	[33]	China	Diet and physical activity	Primary care	3	Group	A year	23
Small Steps for Big Changes	[34, 35]	Canada	Diet and physical activity	Community	2	Individual	Less than a year	6
South Asian Health and Prevention Education Study pilot study	[36]	United States	Diet and physical activity	Not described	2	Group	A year	16
Group Lifestyle Balance in women VA	[37]	United States	Diet and physical activity	Primary care	4	Group	A year	22
PVS-PREDIAPS	[38–40]	Spain	Diet and physical activity	Primary care	9	Not described	Not described	not described
De Por Vida	[41]	United States	Diet and physical activity	Primary care	1	Group	More than a year	32
Help Educate to Eliminate Diabetes	[42]	United States	Diet and physical activity	Community	not described	Group	Less than a year	8
National Diabetes Prevention Program	[43–47]	United States	Diet and physical activity	Primary care + Community	435	Group	A year	22
National Diabetes Prevention Program	[48]	United States	Diet and physical activity	Primary care	8	Group	A year	22
Thai Diabetes Prevention Program	[49]	Thailand	Diet and physical activity	Primary care	68	Group	More than a year	17
Prediabetes Intervention Package	[50–52]	New Zealand	Diet	Primary care + Community	4	Combination of group and individual	Less than a year	10
Diabetes Prevention Program in Large Healthcare Systems Evaluation of a Lifestyle Intervention Adopted for Clinical Practice for Diabetes Prevention	[53, 54]	United States	Diet and physical activity	Primary care	20	Group	A year	22
Diabetes Prevention Program for Chinese American Food Service Employees	[55]	United States	Diet and physical activity	Workplace	1	Individual	Less than a year	12
A Person-Centered Approach to Self-Management and Reciprocal Learning for the Prevention and Management of Type 2 Diabetes	[56]	Other: Uganda, South Africa, Sweden	Uganda: Diet and physical activity and alcohol, smoking, self-care and medication; South Africa: Diet and physical activity and alcohol, smoking, self-care and medication, sexuality and stress management; Sweden: Diet and physical activity	Primary care	Not described	Combination of group and individual	A year	13
Power Up for Health	[57–59]	United States	Diet and physical activity	Community	8	Group	A year	16
Group Lifestyle Balance Program in a Military Setting	[60]	United States	Diet and physical activity	Primary care	1	Combination of group and individual	Less than a year	4
National Health Service Diabetes Prevention Program	[61–71]	United Kingdom	Diet and physical activity	Primary care + Community	41	Group	Less than a year	13
Diabetes Prevention -Transferring Findings from European Research to Society	[72]	Spain	Diet and physical activity	Primary care	103	Combination of group and individual	More than a year	16
Diabetes Prevention Program—Group Lifestyle Balance	[73]	United States	Diet and physical activity	Community	3	Group	A year	22
Kerala Diabetes Prevention Program	[74–79]	India	Other: Diet, physical activity, smoking, alcohol, sleep	Community	30	Group	A year	15
Promotora Effectiveness Versus Metformin Trial	[80]	United States	Other: Diet, physical activity, and medication intake	Primary care	1	Group	A year	24
Sydney Diabetes Prevention Program	[81]	Australia	Diet and physical activity	Primary care	1	Combination of group and individual	A year	7
Weight watchers	[82]	United Kingdom	Diet and physical activity	Community	Not described	Group	Less than a year	48
Intermountain Healthcare Diabetes Prevention Program	[83–85]	United States	Diet and physical activity	Workplace	63	Combination of group and individual	Less than a year	12
National Diabetes Prevention Programs in Los Angeles	[86–89]	United States	Diet and physical activity	Primary care + Community	4	Group	A year	24
Veterans Affairs Diabetes Prevention Program vs. MOVE!	[90–92]	United States	Diet and physical activity	Primary care	3	Group	A year	19 (MOVE) 22 (VADPP)
Primary Care Diabetes Prevention Program	[93]	Canada	Diet and physical activity	Primary care	6	Group	A year	Not described
Sydney Diabetes Prevention Program	[94]	Australia	Diet and physical activity	Primary care	83	Combination of group and individual	A year	8
Diabetes Prevention Program for Diabetes Risk Reduction in Chinese Immigrants in New York City	[95]	United States	Diet and physical activity	Community	1	Combination of group and individual	A year	18
SLIM iMplementation Experience Region Noord- en Oost-Gelderland	[96–99]	The Netherlands	Diet and physical activity	Primary care	49	Combination of group and individual	Less than a year	10
YMCA Diabetes Prevention Program	[100]	United States	Diet and physical activity	Community	2	Group	A year	25
National Diabetes Prevention Program within a Health Disparities Population	[101]	United States	Diet and physical activity	Community	3	Group	A year	16
Weight Watchers vs. Your Game Plan to Prevent Type 2 Diabetes	[102]	United States	Diet and physical activity	Community	Not described	Group	A year	Unlimited
GLICEMIA	[103]	Germany	Diet and physical activity	Primary care	42	Combination of group and individual	A year	8
Intervention Program in a Large Israeli Healthcare Organization with an Emphasize on Mediterranean Diet (MedDiet) and Physical Activity	[104]	Israel	Diet and physical activity	Primary care	Not described	Combination of group and individual	Less than a year	10 group meetings or 8 individual meetings over 6-months
Road Map Towards Diabetes Prevention	[105]	The Netherlands	Diet and physical activity	Primary care	26	Individual	A year	5
Reaching Out and Preventing Increases in Diabetes	[106, 107]	United States	Diet and physical activity	Primary care + Community	15	Group	A year	25
Promotora-Led Diabetes Prevention Program (PL-DPP) in Latinas	[108]	United States	Diet and physical activity	Community	2	Group	A year	24
Healthy Living Program (HLP) and a Diabetes Prevention Program (DPP)	[109]	United States	Diet and physical activity	Primary care + Community	4	Combination of group and individual	Less than a year	24
New Life, New You	[110]	United Kingdom	Diet and physical activity	Community	1	Group	Less than a year	8
Group Lifestyle Balance Program Delivered by Diabetes Educators	[111, 112]	United States	Diet and physical activity	Primary care	3	Group	A year	21
Church-based Diabetes Prevention Program	[113]	United States	Diet and physical activity	Community	1	Combination of group and individual	Less than a year	12
New York State YMCA Diabetes Prevention Program	[114, 115]	United States	Diet and physical activity	Primary care + Community	14	Group	A year	22
Reaching Immigrants through Community Empowerment	[116]	United States	Diet and physical activity	Community	Not described	Combination of group and individual	Less than a year	16
Special Diabetes Program for Indians Diabetes Prevention Program	[117, 118]	United States	Diet and physical activity	Primary care	36	Combination of group and individual	Less than a year	16
New Life, New You	[119]	United Kingdom	Diet and physical activity	Community	Not described	Group	Less than a year	20
DHIAAN	[120–122]	The Netherlands	Diet and physical activity	Primary care	Not described	Combination of group and individual	A year	32
YMCA Diabetes Prevention Program	[123]	United States	Diet and physical activity	Community	46	Group	A year	24
Lifestyle intervention programs in the Brazilian public health system	[124]	Brazil	Diet and physical activity	Primary care	1	Combination of group and individual	Less than a year	13
‘An Ounce of Prevention’ Healthy Lifestyle and Diabetes Program	[125]	Canada	Diet and physical activity	Primary care	1	Combination of group and individual	Less than a year	17
NHS Bolton (face-to-face)	[126]	United Kingdom	Diet and physical activity	Primary care	15	Individual	Less than a year	Not described
Power to Prevent (P2P): A Family Lifestyle Approach to Diabetes Prevention	[127]	United States	Diet and physical activity	Community	3	Group	Less than a year	12
Melbourne Diabetes Prevention Study	[128–130]	Australia	Diet and physical activity	Primary care	Not described	Combination of group and individual	Less than a year	6
Health is Wealth	[131]	United States	Diet and physical activity	Community	1	Group	Less than a year	8
Lawrence Latino Diabetes Prevention Project	[132]	United States	Diet and physical activity	Primary care	1	Combination of group and individual	A year	16
Diabetes in Europe- Prevention Using Lifestyle, Physical Activity and Nutritional Intervention Project	[133]	Austria	Diet and physical activity	Primary care	Not described	Group	A year	5
Montana cardiovascular disease and diabetes prevention program	[134–139]	United States	Diet and physical activity	Primary care + Community	39	Combination of group and individual	Not described	N/A
Making the Connection—Healthy Living Program	[140]	United States	Diet and physical activity	Community	Not described	Group	A year	22
DPP in Thailand	[141]	Thailand	Diet and physical activity	Primary care + Community	Not described	Group	Less than a year	Not described
DPP in African American Churches	[142]	United States	Diet and physical activity	Community	5	Group	A year	6 and 16 (two arms)
Reflections on Progress of DPP in Australia. Life! Taking Action on Diabetes	[143, 144]	Australia	Diet and physical activity	Primary care	Not described	Group	Less than a year	6
Diabetes in Europe Prevention using Lifestyle, Physical Activity and Nutritional Intervention	[145, 146]	Poland	Diet and physical activity	Primary care	9	Group	Less than a year	17
Live Well, Be Well Study	[147–149]	United States	Diet and physical activity	Community	4	Combination of group and individual	A year	19
Diabetes in Europe Prevention using Lifestyle, Physical Activity and Nutritional Intervention	[150]	Greece	Diet and physical activity	Primary care + Workplace	12	Group	A year	6
Diabetes Prevention Program in YMCAs	[151]	United States	Diet and physical activity	Community	Not described	Group	A year	N/A
Church-Based Diabetes Prevention Programs	[152]	United States	Diet and physical activity	Community	Not described	Group	Not described	Not described
Diet-Exercise-Activity-Lifestyle Program	[153]	United States	Diet and physical activity	Primary care	1	Combination of group and individual	A year	5
Active Prevention in High Risk Individuals of Diabetes Type 2 in and around Eindhoven	[154–157]	The Netherlands	Diet and physical activity	Primary care	14	Combination of group and individual	More than a year	17
Beweegkuur	[158, 159]	The Netherlands	Diet and physical activity	Primary care	18	Individual	A year	unknown and individually tailored
Healthy-Living Partnerships to Prevent Diabetes	[160–162]	United States	Diet and physical activity	Community	1	Combination of group and individual	A year	28–45
Living on the Edge of Diabetes	[163]	United States	Diet and physical activity	Community	1	Combination of group and individual	A year	4
Translating the Diabetes Prevention Program	[164]	United States	Diet and physical activity	Primary care	6	Group	Less than a year	12
National Type 2 Diabetes Prevention Programme in Finland	[165–168]	Finland	Diet and physical activity	Primary care	400	Combination of group and individual	Less than a year	5
Diabetes Prevention Program in a Rural Africa-American Church	[169]	United States	Diet and physical activity	Community	1	Group	A year	6
Worksite Diabetes Prevention Program	[170, 171]	United States	Diet and physical activity	Workplace	1	Combination of group and individual	A year	22
Lifestyle Intervention Program for Maori	[172]	New Zealand	Diet and physical activity	Community	1	Combination of group and individual	Less than a year	4
National Diabetes Prevention Programme—Hawaii	[173]	United States	Other: diet, physical activity, and stress management	Primary care	7	Group	A year	22
Prevention of Diabetes in Euskadi	[174, 175]	Spain	Diet and physical activity	Primary care	14	Group	A year	4
Diabetes Education & Prevention with a Lifestyle Intervention Offered at the YMCA (DEPLOY)	[176–178]	United States	Diet and physical activity	Community	1	Group	A year	22
Tawazon	[179]	Saudi Arabia	Diet and physical activity	Primary care	4	Group	Less than a year	22
South Gloucestershire Diabetes Prevention (Pilot) Programme	[180]	United Kingdom	Diet and physical activity	Primary care	2	Group	Less than a year	6
Life in BALANCE	[181]	United States	Diet and physical activity	Community	1	Group	Less than a year	16
Good Ageing in Lahti Region	[182, 183]	Finland	Diet and physical activity	Primary care	16	Group	Less than a year	6
Norfolk Diabetes Prevention Study	[184–186]	United Kingdom	Diet and physical activity	Primary care	8	Group	More than a year	21
STAR Family Health Team (FHT) Prediabetes Lifestyle Intervention Program	[187]	Canada	Diet and physical activity	Primary care	1	Group	Less than a year	8
Living Well Taking Control	[188]	United Kingdom	Diet and physical activity	Community	4	Combination of group and individual	A year	10
Health Under Construction	[189]	The Netherlands	Diet and physical activity	Workplace	25	Individual	Not described	7
Diabetes Prevention Program Using Primary Care Nurses	[190, 191]	United States	Diet and physical activity	Primary care	5	Individual	Less than a year	11
DPP within General Practice in Denmark	[192]	Denmark	Diet and physical activity	Primary care	8	Individual	A year	6
Diabetes in Europe Prevention using Lifestyle, Physical Activity and Nutritional	[193]	Spain	Diet and physical activity	Primary care	16	Combination of group and individual	Less than a year	4
DPP in Norfolk	[194]	United Kingdom	Diet and physical activity	Primary care + Community	Not described	Group	Not described	4
YMCA Diabetes Prevention Program	[195]	United States	Diet and physical activity	Community	N/A	Group	A year	22

The number of sites varied from small single site pilot/feasibility studies to national implementation efforts. Most programs (*n* = 52, 56%) presented data from less than 10 sites, while a minority of programs (*n* = 3, 3%) had greater than 100 sites. Programs were group-based (*n* = 53, 56%), a combination of group and individual (*n* = 32, 34%) or individual (*n* = 8, 9%), with one (1%) not describing the type of program. Most programs included healthcare professionals in some capacity (*n* = 68, 72%).

### Implementation strategies

 A total of 69 of the 73 ERIC implementation strategies were retrospectively extracted (M = 13.8 ± 9.1, range 0 – 41 per program). The most reported strategies were: *conduct educational meetings, build a coalition,* and *promote adaptability* (see Table 2 for overview of frequency data per strategy). Four strategies were not represented within the data: *change liability laws, develop disincentives, make billing easier*, and *start a dissemination organization*. The strategies were extracted from each individual study (*N* = 166), then consolidated according to program (n = 94). The highest number of strategies were identified in large implementation scale-up efforts (United Kingdom National Health Service DPP, U.S. National DPP, and the Kerala DPP in India; 41, 39, 34 strategies respectively). One study did not contain any identified strategies (see Additional file 3 for extracted implementation strategies identified per program). Two studies reported ERIC strategies within the published manuscript [38, 48]. In both studies, the final set of implementation strategies extracted from this review differed from the implementation strategy list published within the manuscript.

**Table 2:** Frequency of implementation strategies represented within programs (*n* = 94)

Implementation Strategy	Frequency represented (%)
Build a coalition	64 (68%)
Conduct educational meetings	63 (67%)
Promote adaptability	54 (57%)
Prepare patients/consumers to be active participants	46 (49%)
Increase demand	43 (46%)
Use mass media	40 (43%)
Obtain and use patients/consumers and family feedback	39 (41%)
Develop and organize quality monitoring systems	35 (37%)
Conduct local consensus discussions	34 (36%)
Develop academic partnerships	34 (36%)
Intervene with patients/consumers to enhance uptake and adherence	34 (36%)
Inform local opinion leaders	29 (31%)
Use advisory boards and workgroups	29 (31%)
Develop and implement tools for quality monitoring	28 (30%)
Stage implementation scale-up	28 (30%)
Promote network weaving	27 (29%)
Distribute educational materials	26 (28%)
Purposefully reexamine the implementation	26 (28%)
Develop educational materials	25 (27%)
Make training dynamic	25 (27%)
Conduct local needs assessment	23 (27%)
Develop resource sharing agreements	23 (24%)
Use data warehousing techniques	23 (24%)
Provide ongoing consultation	22 (24%)
Conduct educational outreach visits	20 (21%)
Alter patient/ consumer fees	19 (20%)
Conduct ongoing training	19 (20%)
Change physical structure and equipment	18 (19%)
Facilitation	17 (18%)
Access new funding	16 (17%)
Change record systems	16 (17%)
Create new clinical teams	16 (17%)
Identify and prepare champions	16 (17%)
Revise professional roles	16 (17%)
Create a learning collaborative	14 (15%)
Fund and contract for the clinical innovation	14 (15%)
Provide clinical supervision	14 (15%)
Work with educational institutions	14 (15%)
Audit and provide feedback	13 (14%)
Change service sites	13 (14%)
Create or change credentialing and/or licensure standards	13 (14%)
Organize clinician implementation team meetings	13 (14%)
Tailor strategies	13 (14%)
Assess for readiness and identify barriers and facilitators	12 (13%)
Use train-the-trainer strategies	12 (13%)
Centralize technical assistance	11 (12%)
Develop a formal implementation blueprint	11 (12%)
Obtain formal commitments	11 (12%)
Recruit, designate, and train for leadership	11 (12%)
Involve patients/ consumers and family members	10 (11%)
Involve executive boards	8 (9%)
Visit other sites	7 (7%)
Alter incentive/ allowance structures	6 (6%)
Conduct cyclical small tests of change	6 (6%)
Place innovation on fee for service lists/formularies	6 (6%)
Provide local technical assistance	6 (6%)
Change accreditation or membership requirements	5 (5%)
Develop an implementation glossary	5 (5%)
Use data experts	5 (5%)
Facilitate relay of clinical data to providers	4 (4%)
Shadow other experts	4 (4%)
Use other payment schemes	4 (4%)
Model and simulate change	3 (3%)
Remind clinicians	3 (3%)
Capture and share local knowledge	2 (2%)
Identify early adopters	2 (2%)
Mandate change	2 (2%)
Use an implementation advisor	2 (2%)
Use capitated payments	2 (2%)
Change liability laws	0 (0%)
Develop disincentives	0 (0%)
Make billing easier	0 (0%)
Start a dissemination organization	0 (0%)

The count shows the number of programs (representing a group of manuscripts) that authors identified the presence of an implementation strategy

Authors of the ERIC compilation published strategy clusters and quadrants of importance and feasibility [28]. The most frequently represented thematic cluster was *develop stakeholder interrelationships* and *train and educate stakeholders* (represented in 86% and 81% of programs respectively), while the least frequently represented clusters were: *utilize financial strategies, change infrastructure, support clinicians,* and *provide interactive assistance* (represented in 44%, 51%, and 50% of programs respectively; see Table 3). Similar results are observed when looking at the number of programs represented within each thematic cluster. Overall, there were more strategies within the high importance, high feasibility quadrant than the rest of the quadrants. All quadrants had good representation amongst the programs (80–91%; See Table 4).

**Table 3:** Overview of thematic clusters represented within this review

Cluster name	Frequency the thematic cluster represented	Total # (%) of programs represented within cluster	# of strategies within cluster	Range of strategies represented within cluster
Develop Stakeholder Interrelationships	295	81 (86%)	17	0–11
Train and Educate Stakeholders	254	76 (81%)	11	0–8
Use evaluative and iterative strategies	227	68 (72%)	10	0–10
Engage Consumers	169	73 (78%)	5	0–5
Adapt and Tailor to the Context	93	74 (79%)	4	0–3
Utilize Financial Strategies	67	41 (44%)	9	0–6
Change infrastructure	66	60 (64%)	8	0–4
Support Clinicians	60	48 (51%)	5	0–3
Provide Interactive Assistance	49	47 (50%)	4	0–4

Thematic strategy clusters as defined from [28]

**Table 4:** Overview of importance and feasibility quadrants represented within this review

Zone name	Frequency the zone represented	Total # (%) of programs represented within zone	# of strategies represented within zone	Range of strategies represented within zone
High Importance and High Feasibility	634	86 (91%)	28	0–20
Low Importance and Low Feasibility	300	84 (89%)	24	0–11
Low Importance and High Feasibility	189	80 (85%)	12	0–6
High Importance and Low Feasibility	157	75 (80%)	9	0–7

Importance and feasibilities quadrants as defined from [28]

### Implementation determinants and outcomes

Most programs reported program effectiveness data (*n* = 76, 81%) with less focus on implementation data (*n* = 37, 39%). Twenty percent of programs (*n* = 19) reported implementation outcomes (4% [*n* = 4] at both the program and implementation strategy level, and 17% [*n* = 16] at only the program level) and 20% of programs (*n* = 19) reported implementation determinants (20% [*n* = 19] at both the program and implementation strategy level, 2% [*n* = 2] at only the program level, and 1% [*n* = 1] at only the implementation strategy level).^1^ A minority of studies reported coach or site descriptive details (*n* = 22, 23%). Most programs reported data at the participant level (*n* = 83, 88%), with less focus on the facilitator level (*n* = 30, 32%), site level (*n* = 17, 18%), cost data (*n* = 15, 16%), managerial level (*n* = 9, 10%), organizational level (*n* = 6, 6%), community level (*n* = 1, 1%) and a small number of programs reported a descriptive overview of reflections on the implementation effort (*n* = 11, 12%). Of the identified levels of data reporting (such as participant, site, facilitator etc.), programs reported one level (*n* = 51, 54%), two levels (*n* = 18, 19%), 3 levels (*n* = 13, 14%), four levels (*n* = 7, 7%), five levels (*n* = 4, 4%) and one program reported six levels (*n* = 1, 1%).

### Theoretical frameworks

Most studies lacked an implementation theory, model, or framework (*N* = 131, 79%). Studies used implementation theories, frameworks, or models to inform analysis (*N* = 17, 49%), design (*N* = 12, 34%) or data collection (*N* = 12, 11%). Two studies (6%) used two different implementation frameworks for a combination of purposes (e.g., one for data collection and one for analysis). When classified according to the categories by Nilsen et al. [29], the majority were used for evaluation (*N* = 15, 43%) and included using the Reach, Effectiveness, Adoption, Implementation, Maintenance framework (REAIM); Implementation outcomes framework; penetration, implementation, participation, and effectiveness (PIPE); Process evaluation for public health interventions and research; and the Precede- Proceed model. Determinant theories, models or frameworks were used 14% (*n* = 5) of the time and included the Consolidated Framework for Implementation Research (CFIR) and Theoretical Domains Framework (TDF). Process theories, models or frameworks were used 11% (*n* = 4) of the time and included the Knowledge to Action Framework (K2A); Medical Research Council framework (MRC), Intervention Mapping, and the process for adapting evidence–based behavioral interventions for new settings and target populations by McKleroy. Classic theories, models or frameworks were used 20% (*n* = 7) of the time and included the National Institute of Health Behaviour Change Consortium (NIH BCC; *n* = 4); social network analysis; social learning theory, and community-based participatory research (CBPR). Implementation theories, models or frameworks were used 6% (*n* = 2) of the time and included the Capability, Opportunity, Motivation – Behaviour model (COM-B) and the Health care intervention implementation theory. Finally, two studies (6%) used two different implementation frameworks for a combination of categories (e.g., one for process and one for evaluation). A few theories were represented within multiple studies; REAIM (*n* = 12), CFIR (*n* = 3), TDF (*n* = 3), and the National Institute for Health Behaviour Change Consortium (*n* = 4).

## Discussion

This review explored the implementation strategies used and implementation variables reported from DPPs conducted in clinical practice and community settings. To optimally build the field of implementation science, greater understanding of how evidence-based programs are implemented (i.e., identification of implementation strategies), how well the implementation proceeded (i.e., reporting of implementation outcomes) and under what context (i.e., reporting of implementation determinants) is needed. This comprehensive systematic search and review extended the work of previous reviews by reporting on worldwide in-person DPPs, including projects at varying stages of implementation/scale-up, and extracting data using standardized tools [10, 17]. Laying the foundation for future work, this review is the first of its kind to retrospectively identify implementation strategies in DPPs implemented in the clinical practice and community settings.

### Retrospective extraction of implementation strategies

Overall, after reviewing each study and retrospectively extracting implementation strategies, we identified 69 out of 73 ERIC strategies. On average, programs used 13.7 strategies, falling slightly below the range reported from other projects examining implementation strategies (range 16 – 59; [196–199]). This discrepancy may be attributed to retrospective coding resulting in fewer identified strategies compared to prospective coding. Retrospective coding relies solely on information available within the manuscript and may not capture all strategies present during the implementation process [200]. A range of strategies (0–41) was documented. Small single-site studies tended to focus on demonstrating effectiveness within a new setting and yielded fewer strategies. In contrast, larger implementation projects with multiple sites tended to yield more strategies. Large, multi-site projects may require greater planning and selection of strategies to support implementation and consistency across sites. In addition, the data revealed trends that large multi-site implementation projects typically had a greater focus on implementation, resulting in more detailed reporting of implementation variables and strategies and ultimately the extraction of more implementation outcomes, determinants, and strategies. Finally, the more manuscripts contained within a program grouping (often correlated with the size of the implementation project), the more implementation strategies identified. Having numerous publications may have enabled greater depth and breadth of the research while providing more space for detailed reporting and subsequently increasing the number of extracted strategies. Regardless of the project’s size, selecting and reporting implementation strategies is important. Thoughtfully designed pilot and feasibility studies are needed to adequately prepare and test implementation strategies for larger trials and sustainable community-based DPPs [201].

The most common strategies extracted were *conduct educational meetings* (*n* = 64), *build a coalition* (*n* = 63), and *promote adaptability* (*n* = 54). In a previous review conducted in implementing community-based chronic disease prevention programs, *conduct educational meetings* was the most common strategy reported [202]. However, authors used a previous (shorter) version of the ERIC compilation and results may not be comparable to the current review. Our results differ compared to a publication that specified implementation strategies within seven large implementation interventions [196]. Thirty-three strategies were identified yet both *build a coalition* and *promote adaptability* were not represented [196]. The most common strategies from the seven implementation interventions were: *develop educational materials, distribute educational materials, facilitate implementation, assess for readiness and identify barriers and facilitators, develop an implementation blueprint*, and *audit and provide feedback* [196]. The varied findings highlight the need for more research and reporting of implementation strategies to better inform recommendations on what strategies to use, when and why. For example, early research suggests a potential correlation between the number of strategies used and the uptake/delivery of an evidence-based innovation [199]. However, scenarios exist where simple implementation strategies may be more effective than complex, multicomponent ones [203]. The type of strategy (e.g., active strategies, sustaining strategies, and tailoring strategies to the individual context) may be more important than the quantity [203]. Furthermore, there could be a temporal or project size connection associated with strategy use. Some strategies may be more important in the planning phase of implementation compared to active implementation or project sustainability. Similarly, some strategies may be more important for multi-site projects compared to single-site projects. All these reasons could have led to the varied representation of strategies in the literature. Nonetheless, current results highlight commonly used strategies from a large sample of DPPs. Results can be used to help inform the design of future implementation projects. Future research is needed to further understand the selection and effectiveness of strategies, potentially as strategies relate to the size and stage of the project.

When examining the thematic clusters of strategies [28] from this review’s results, *engaging consumers* was the most represented cluster, followed by *use of evaluative and iterative strategies, adapt and tailor to the context*, and *train and educate stakeholders* (see Table 3 for overview of cluster representation). Results are unable to be compared to previous research as no prior research reported thematic clusters. Not surprisingly, the most represented thematic cluster is at the participant (i.e., consumer) level suggesting that most implementation strategies target the participant experience. Thematic clusters that support staff facilitating the implementation (e.g., *provide interactive assistance* and *support clinicians*) had limited representation. Support staff and/or program facilitators have vital roles in the execution of a program and implementation strategies and must be supported to ensure smooth operation of a project. Strategies that support staff are important to build capacity for implementation as education and training may not be sufficient [204]. Research from the behaviour change field supports this observation as often education is insufficient to change behaviour and additional behaviour change techniques to support individuals are needed [205, 206]. The least represented clusters include *utilize financial strategies* and *change infrastructure*. These two clusters may be more difficult to change. Indeed, almost all the strategies in these two clusters are found in quadrant I, representing low feasibility and low importance [28]. More research is needed to understand the relationship between specific strategies and/or clusters of strategies and the success of innovation uptake.

### Reporting implementation details

Overall, reporting implementation details was poor. Explicitly reporting ERIC implementation strategies in DPPs was rare. Only two of the 93 included programs reported implementation strategies using definitions provided by ERIC. This low reporting of implementation strategies is consistent with other fields [196]. Although these two articles reported greater implementation details, the articles still fell short of meeting Proctor and colleagues reporting recommendations released in 2013 [11]. In 2017, the Standards for Reporting Implementation Studies (StaRI) statement and checklist was published specifically requesting strategies be reported [207]. Despite the current review including articles published after the release of StaRI, only two publications reported strategies [38, 48]. Greater uptake and enforcement of reporting guidelines are needed.

Similarly, despite all included programs being implemented in clinical practice and community settings, only a minority of programs reported data from viewpoints other than program recipients. Program facilitators and support staff have an integral role in a program’s effectiveness in clinical practice and community settings. Research conducted in clinical practice and community settings, and specifically implementation research, must consider a broader interpretation of ‘participants’ [18]. Multiple layers of staff (facilitators, managers, administrative staff, leadership) support implementation and they all act within a broader environment (site, organization, community) that impacts implementation. Unfortunately, only 23% of programs reported descriptive data on staff or the site(s). Implementation research must improve at reporting crucial contextual information that extends beyond the program and program recipients.

Results affirm that there is a higher frequency of reporting program effectiveness data compared to implementation outcomes or determinants. When implementation outcomes are reported, they are most often at the program level (e.g., program fidelity), with little to no attention to the implementation strategy level (e.g., implementation strategy fidelity). This was expected as research at the implementation strategy level is lacking and difficult to operationalize and measure. For example, research on fidelity to an implementation strategy is improbable unless researchers are aware of, and have reported, implementation strategies. When implementation determinants were reported, both program level (e.g., acceptability of the program) and implementation strategy level (e.g., acceptability of the implementation strategies) were represented. Representation of both levels may be because implementation determinant data was often collected by qualitative interview or focus group data, allowing for easier flexibility and range of questions. Overall, there is a need for greater focus on implementation determinants and outcomes.

Lastly, only 25% of studies included an implementation theory, framework, or model. Framework classifications align with the pattern observed in reported variables. For example, studies reporting reach were informed by the RE-AIM framework (evaluation classification); studies reporting implementation determinants were often informed by determinant frameworks. Thus, continued advocacy for implementation research is needed which may in turn increase the use of implementation theories, models or frameworks to increase reporting of implementation variables.

### Challenges with the ERIC compilation of implementation strategies

There were many challenges with extracting implementation strategies. Some strategies are inherently linked to other strategies [11]. For example, the provision of technical assistance, can be coded as either *provide local technical assistance* or *centralize technical assistance* depending on where the assistance is coming from. In addition, the action of providing technical assistance may include a range of other implementation strategies such as *provide clinical supervision, provide ongoing consultation, facilitate, visit other sites, develop a learning collaborative, conduct educational meetings, develop education materials,* and *distribute educational materials* [208]. In other instances, an implementation strategy directly implies multiple actions. For example, *audit and feedback* requires three discrete steps: data collection, review of data (audit), and provision of feedback. The data needed for an audit may have been collected as part of other implementation strategies (e.g., *develop and implement quality monitoring tools* and *develop and implement quality monitoring systems*). Situations could arise where audit occurs without feedback, which limits the codability of this strategy. Finally, the provision of feedback can occur in a variety of ways from passive strategies such as a report, to active strategies, such as individual one-on-one feedback meetings. Until strategies are disentangled from one another, coding will continue to be difficult. Indeed, the authors of ERIC recommend to always describe the discrete strategies within a larger strategy [11]. Detailed reporting is needed to understand exactly what was done, and implementation strategies should represent irreducible actions. The challenges experienced with the ERIC tool, and poor state of reporting in the included articles, reinforce the need for authors to provide details on the actor, action, action target, temporality, dose, implementation outcome affected, and justification [11]. These details will help other researchers more clearly understand how the strategy was enacted.

Discrepancies were found between our interpretation of the strategy definitions and the examples reported by authors that explicitly reported ERIC implementation strategies [38]. Despite research demonstrating that implementation strategies are conceptually distinct [28] when used in practice, reporting the correct strategies may be difficult. Similar to coding behaviour change techniques, additional supports such as standardized training, mobile phone application, and a technical assistance website may be needed to support researchers in accurately selecting strategies to ensure consistency in reporting [209]. Ambiguity in the coding of implementation strategies undermines future efforts to collate research. Before we can understand what strategies are important, we must first understand what strategies have been used through accurate and consistent reporting [197]. This review makes the first steps in identifying strategies used within DPPs, using consensus-based retrospective coding on a large sample of programs.

We have suggestions to improve the ERIC compilation. Many strategies had a clinical focus and may benefit from more general terms. For example, *organize clinician implementation team meetings* specifies a profession; however, other roles (e.g., nurses, fitness staff) may implement initiatives. While many programs emphasized dissemination, none created a specific organization, so we could not code the strategy *start a dissemination organization*. Since dissemination is a standard part of the research process, we recommend renaming the strategy to better reflect the common practice. We partially agree with the suggestions by Bunger and colleagues [197]. Their suggestion to add *obtain worker feedback about implementation plan* could already be coded under *conduct local consensus discussions*. However, we felt that a strategy to *obtain staff feedback on implementation* would be a helpful addition (note we changed ‘worker’ to ‘staff’ for generalizability and removed the word plan). Similarly, their suggestion to add *plan for outcome evaluation* may already be encompassed in *develop a formal implementation blueprint*. The definition for *develop a formal implementation blueprint* could be enhanced by revising the wording ‘appropriate performance/progress measures’ to specifically highlight both outcome evaluation and implementation evaluation. We recommend three additional strategies: *discuss implementation among leadership staff from site(s), training for the support team*, and *obtain feedback from support team*. Support staff and leadership staff have important roles within implementation efforts [204] and feedback, training, and meetings should be occurring beyond just the individuals facilitating the program as is currently implied in existing ERIC strategies. We disagree with two strategy suggestions by Perry and colleagues [196]. Specifically, *engage community resources* could be coded under *develop resource sharing agreements*, while *create online learning communities* could be coded under *create a learning collaborative*. We agree with the Perry and colleagues’ [196] addition of *assess and redesign workflow.* Assessing and building on existing workflows were mentioned as positive implementation strategies within the included programs [210–212]. There is clearly room to improve the strategies and their definitions for greater consistency and clarity.

### Strengths, limitations and future directions

Extracting implementation strategies was limited to retrospective coding of published studies and to the amount of detail reported. Retrospective coding may underreport strategies when compared to the benefits of tacit knowledge held by authors when prospectively reporting strategies [200]. It is difficult to disentangle strategies implemented for research purposes (e.g., free program, evaluation or reporting strategy) that may or may not remain after the research has concluded. The research team encountered difficulties applying ERIC definitions to extract strategies. To establish coding reliability, a codebook was created to assist consistent application of definitions, all manuscripts were coded by two researchers, and numerous conversations were held to establish consensus. A possibility exists that our interpretation of a strategy was incorrect. Readers may review our codebook (Additional file 2) to understand our interpretation of ERIC definitions and coding decisions/rules. Future research is needed to refine the ERIC tool including common strategy groupings, revisions to ERIC labels and/or coding rules for related strategies. The codebook provided may help inform this work. We did not extract individual implementation outcomes and determinants due to the lack of meaningful data coupled with the narrow definitions provided the implementation dataset [17]. Overall, the conclusion remains the same, there are significant gaps in reporting implementation level determinants and outcomes. In addition, we extracted any theory, model, or framework explicitly mentioned in an included study and used for implementation research purposes. However, we did not access to what extent the theory, model, or framework was used nor the quality of use.

The screening process had the potential for bias, which may have resulted in the omission of relevant articles. Additionally, the data collected from this systematic search and review has noted limitations (i.e., retrospective coding of published manuscripts, exclusion of digital DPPs). We acknowledge that additional relevant studies may have been published since our search. However, we do not expect these to significantly affect our conclusions given the large initial dataset and the few additional relevant articles. We conducted a search on MEDLINE in March 2025 and found only one relevant article that explicitly reported ERIC implementation strategies [204]. There remains a large focus on effectiveness data (e.g., [210]) with implementation data often detailed in descriptive qualitative manuscripts focused on lessons learned (e.g., [211, 212]). Despite the limitations, the large dataset contains interesting data and can inform future directions. An identified trend of having more strategies extracted from larger implementation projects compared to smaller projects is worth exploring. There may be strategies that become more important as a project increases in size and maturity [213]. In multi-site projects, understanding the fidelity of implementation strategy enactment could uncover reasons for differences in implementation success between sites while uncovering temporal enactment of strategies.

## Conclusions

Strong evidence supports DPPs as a potent method to combat the rising prevalence of T2D, yet research on how to implement DPPs is lacking. To our knowledge this was the first review that specifically extracted implementation strategies, outcomes, and determinants from previously reported DPPs implemented in a clinical practice and community setting. Extracting implementation strategies was difficult due to a lack of detailed reporting and difficulty applying the ERIC definitions. Most DPPs implemented within a clinical practice and community setting have focused on reporting participant level program effectiveness data with a lack of implementation level outcomes and determinants. Without implementation level data it is difficult to understand whether programs have been implemented effectively and how and why the implementation was or was not effective. Greater focus on implementation is needed to build the evidence base for evidence-based implementation.

## Supplementary Information

Supplementary Material 1.

Supplementary Material 2.

Supplementary Material 3.

## Data Availability

The datasets used and/or analysed during the current study are available from the corresponding author on reasonable request.

## Abbreviations

CFIR: Consolidated Framework for Implementation Research
COM-B: Capability, Opportunity, Motivation – Behaviour model
DPP: Diabetes Prevention Program
ERIC: Expert Recommendations for Implementing Change project’s Compilation of Implementation Strategies
K2A: Knowledge to Action Framework
MRC: Medical Research Council
NIH BCC: National Institute of Health Behaviour Change Consortium
PRISMA: Preferred Reporting Items for Systematic Reviews and Meta-Analysis
PICOS: Population, intervention, comparison, outcomes and study type
PIPE: Penetration, implementation, participation, and effectiveness
RE-AIM: Reach, Effectiveness, Adoption, Implementation, Maintenance framework
STARI: Standards for Reporting Implementation Studies
T2D: Type 2 Diabetes
TDF: Theoretical Domains Framework
U.S.: United States

## Footnotes

1 Note:. Within one program grouping, data on both implementation outcomes and determinants could be reported and at a variety of levels. Implementation outcomes and determinants, as well as the level they are reported at are not mutually exclusive, thus, percentages do not summate to a total percentage.
