# Supplementary material for: Implementation of diabetes prevention programs into clinical practice and community settings: a systematic search and review
Source: Implement Sci Commun. 2025 Jul 9;6:74. doi: 10.1186/s43058-025-00757-2 (PMC12243291; doi:10.1186/s43058-025-00757-2)
Supplement: Supplementary file 2 — Supplementary Material 2. [file 43058_2025_757_MOESM2_ESM.docx]

Codebook and Examples

Note: Purple text are additions made by Tineke to contextualize ERIC definition to the review. Blue hyperlinks are links to other implementation strategies.

Developed from Powell, 2015 [13]

| **Strategy** | **ERIC Definition (Powell, 2015) [15]** | **Ancillary data (Powell, 2015) [15]** | **Contextualized details for review** | **Coding decisions/rules** | **Example(s)** |
| --- | --- | --- | --- | --- | --- |
| Promote adaptability | Identify the ways a clinical innovation (DPP) can be tailored to meet local needs and clarify which elements of the innovation (program) must be maintained to preserve fidelity | Preserving fidelity to the innovation can be an uncertain process if the core elements of the innovation are not empirically defined. | tailoring at the program level. changes made to the program.  e.g., translating participant materials, modifying the number of sessions, change session topics | Do not code if the program was developed that way. There needs to be evidence of a change.  Code only if changes are at the program level. If changes are at the implementation strategy level, consider [tailor strategies](#_Tailor_strategies) | Modify program content for specific ethnic groups |
| Tailor strategies | Tailor the implementation strategies (strategy level) to address barriers and leverage facilitators that were identified through earlier data collection | The tailoring process tends to be idiosyncratic and driven by multiple factors. It is important to identify the core components of the intervention and implementation strategies that are required to maintain fidelity and effectiveness, and to distinguish those components amenable to modification/adaptation. Flottorp et al. [14] and Langley et al. [15] provide guidance regarding many of the multiple factors to consider. Wensing et al. [16, 17] provide an example of a structured approach to tailoring strategies. | tailoring at the organization/ strategy (e.g., training, provider meetings, provision of support, supervision, educational materials for staff etc.) level.  alter strategies or the process based on barriers identified formally through research, examining data collected from site/program and/or tacit knowledge.  After reviewing data, results in a change/addition to an implementation strategy. | If changes are at the program level, consider [promote adaptability](#_Promote_adaptability). Data driven adaptations.  e.g., If there are issues with data entry or referrals review [change record systems.](#_Change_record_systems)  If the strategy is directly tailored from collected data, consider coding any of the following data sources: [obtain and use patients/consumers and family feedback](#_Obtain_and_use); [obtain and use worker feedback](#obtainanduseworkerfeedback); [plan for outcome evaluation](#Planforoutcomeevaluation); [develop and implement tools for quality monitoring](#_Develop_and_implement); [conduct local needs assessment](#_Conduct_local_needs). | After reviewing data from front-line staff, decision to implement ongoing training with a booster session every month. |
| Use data experts | Involve, hire, and/or consult experts to inform management on the use of data generated by implementation efforts | Consider engaging data experts early in the implementation planning process. | Can be internal or external data personnel. | If this individual helps with developing/ integrating or managing the input and sharing data from multiple sources across multiple sites code: [use data warehousing techniques](#_Use_data_warehousing). If this individual also provides expertise on modifications to the record system, code [change record systems.](#_Change_record_systems) | Hire a health information technologist to manage data collection, cleaning, organization, database creation and processes. |
| Use data warehousing techniques | Integrate clinical records across facilities and organizations to facilitate implementation across systems | Records that include variables that can serve as outcome measures are particularly useful. When outcomes of interest are not available, it may be useful to examine proxy measures. | Merging of multiple databases, data protection and security, creating a new database to hold multi-site data. Making it easier to share and record data across multiple organizations/ sites. Participant registry, dashboard. | If the program hired someone specific to integrate/develop/ manage the database, code: [Use data experts](#_Use_data_experts)  If modifications are made to the record system, code [change record systems](#_Change_record_systems).  If modifications are made specifically to facilitate quality monitoring or outcome evaluation, code: [develop and organize quality monitoring systems](#_Develop_and_organize) or [plan for outcome evaluation](#Planforoutcomeevaluation) | Set-up a system that all data collected at each DPP site is entered into a central database. |
| Change accreditation or membership requirements | Strive to alter accreditation standards so that they require or encourage use of the clinical innovation. Work to alter membership organization requirements so that those who want to affiliate with the organization are encouraged or required to use the clinical innovation | None | Accreditation is an assessment against standards to demonstrate competence to carry out the program. To officially recognize, accept or approve sites. | For site/organizational level and not provider level. If at provider level, see: [Create or change credentialing and/or licensure standards](#_Create_or_change) | NDPP uses the DPRP to standardize the DPP across the US, and ensures each new site meets DPP standards. |
| Change liability laws | Participate in liability reform efforts that make clinicians more willing to deliver the clinical innovation | Liability reform can also make clinicians less willing to deliver alternatives to the clinical innovation. | Make changes to laws/contracts for providers |  |  |
| Change physical structure and equipment | Evaluate current configurations and adapt, as needed, the physical structure and/or equipment (e.g., changing the layout of a room, adding equipment) to best accommodate the targeted innovation | None | Purchase new equipment, expand clinic space. Includes the use of new mhealth technology. | If opening a new site review code: [Change service sites](#_Change_service_sites) | Providing new equipment, renting space to accommodate program. |
| Change record systems | Change records systems to allow better assessment of implementation or clinical outcomes | These changes may include modifying the format of progress notes and treatment plans to reflect the innovation (evidence-based practice) being implemented. | Formal modifications to the way data are recorded. Create standardized data collection forms. If multiple settings or data moving outside of the setting, consider data warehousing techniques. | If specific to quality monitoring, see also [Develop and organize quality monitoring systems](#_Develop_and_organize)  If developed a system to link across sites, consider [use data warehousing techniques](#_Use_data_warehousing). If a data expert was hired to help change record systems, consider [use data expert](#_Use_data_experts) | Participants complete questionnaires on iPads. If this is the case, also consider [Change physical structure and equipment](#_Change_physical_structure) |
| Change service sites | Change the location of clinical service sites to increase access | Changing service sites can include collocating different services to better implement complex clinical innovations that require multiple disciplines or services, telemedicine, or bringing the services to the client in their home, the community, or other clinically relevant settings, such as busy public spaces for a client with PTSD. | Open a new site or relocate for better access, e.g., moving diabetes prevention away from the hospital and into the community. | Code if program is implemented in a new setting. If the program is at the same site, but the site had to order new equipment or reorganize, code: [Change physical structure and equipment](#_Change_physical_structure) | Implementing a DPP at a workplace, church to better reach clients. |
| Create or change credentialing and/or licensure standards | Create an organization that certifies clinicians in the innovation or encourage an existing organization to do so. Change governmental professional certification or licensure requirements to include delivering the innovation. Work to alter continuing education requirements to shape professional practice toward the innovation | none | Have a specific organization that provides standardized training for all providers. Or  Have the government formally create new certifications for a new job type (e.g., “lifestyle coach”)  Add as a continuing education credit for professionals to maintain designation. | If at the organizational level (an organization wants to provide the program), see: [Change accreditation or membership requirements](#_Change_accreditation_or) | All staff go through standardized training and receive a certificate upon successful completion. |
| Mandate change | Have leadership declare the priority of the innovation and their determination to have it implemented | It is important to ensure that the individuals mandating the change have the power to do so, as implementers often lack such authority. Working with organizational leadership to develop buy-in and lobby for a change mandate is often needed. It can also be important to inform other stakeholders (e.g., auditors, groups that review services for billing) about the mandate to ensure they are on the same page. | Top-down approach. Leaders at an organization demand the front-line workers to implement the program. | Evidence of a rule, signed agreement, or other form of directive that comes from the top and forces the implementation. |  |
| Start a dissemination organization | Identify or start a separate organization that is responsible for disseminating the clinical innovation. It could be a for-profit or non-profit organization | This strategy can address concerns (e.g., conflict of interest) for situations in which it is desirable to have fidelity monitors that are independent from the care setting. The dissemination organization could be a for-profit or nonprofit organization. The organization could be ‘licensed’ by a university, if the innovation was born within an academic setting. It is important for dissemination organizations to be aware of organizations' approaches to implementing other interventions in order to build upon existing practices. | Dissemination is “to spread” the innovation | Code if the organization or sub-committee sole purpose is dissemination. |  |
| Build a coalition | Recruit and cultivate relationships with partners in the implementation effort | Partnerships can develop around cost-sharing, shared resources, shared training, and the division of responsibilities among partners. This work may proceed naturally from local consensus discussions. Coalition members commonly have defined roles in the implementation effort. | Examine areas that manuscripts mention partnerships to help with funding, sharing resources, training, recruitment, IT help, outreach, dissemination.  The coalition could be an already formed team. | Examine these codes to see if they should also be coded: [develop academic partnerships](#_Develop_academic_partnerships), [local consensus discussions](#_Conduct_local_consensus), [develop resource sharing agreements](#_Develop_resource_sharing)  If members of the coalition sign a contract or MOU, code [obtain formal commitments](#_Obtain_formal_commitments) |  |
| Capture and share local knowledge | Capture local knowledge from implementation sites on how implementers and clinicians made something work in their setting and then share it with other sites | This strategy is often coordinated with centralized technical assistance and learning collaboratives. There are multiple techniques for capturing local knowledge, which could be presented in multiple formats. For example, short YouTube videos could be created that document testimonials from clinicians who have successfully used a given innovation. Another example would be maintaining a running list of a team's response to specific implementation barriers that could be shared readily through a platform like GoogleDocs or Microsoft SharePoint. Additional techniques can be found at [www.liberatingstructures.com](http://www.liberatingstructures.com). | Capture lessons learned and share it with other sites. Program implementation must have started. | Need more than one site to code this strategy.  If only one site, consider [create a learning collaborative](#_Create_a_learning)  If there are formal trips to visit sites, code [visit other sites](#_Visit_other_sites).  If lessons are learned within a formal group of providers/organizations that meet regularly, review [create a learning collaborative](#_Create_a_learning)  If a program is being developed and local opinion are sought consider [conduct local consensus discussion](#_Conduct_local_consensus)  If a centralized individual or organization facilitates the sharing of information, code [centralized technical assistance](#_Centralize_technical_assistance). | Highlight a certain site in a knowledge product (e.g., newsletter/blog/website/spotlight during a monthly meeting) that gets distributed to all sites. The knowledge product reviews site specific lessons learned and how the site overcame barriers. The knowledge product is used to share this information with a goal to help other sites. |
| Conduct local consensus discussions | Include local providers and other stakeholders in discussions that address whether the chosen problem is important (diabetes prevention) and whether the clinical innovation to address it is appropriate (Diabetes Prevention Program) | Identify stakeholders relevant to each project. Further, with each project, there will be a need to identify whether the goal of the consensus discussion is to characterize consensus or build consensus. Utilizing *community based participatory research* (CBPR) principles may be relevant to many innovations. Notably, the chosen problem needs to be a high enough priority, compared to other problems, that attention and resources will be dedicated to addressing the problem. | Initiatives that seek out opinions on relevant local stakeholders (providers, local health authorities, diabetes organizations, participants).  Focus of discussions need to be on the *rationale* for the DPP. Can include prospective participants.  These conversations often occur before implementation, during the planning phase. | Meetings, community presentations, focus groups.  If formal data is collected on the need for the program, facilitators and barriers or other, review [conduct local need assessment](#_Conduct_local_needs) |  |
| Develop academic partnerships | Partner with a university or academic unit for the purposes of shared training and bringing research skills to an implementation project | HIPAA, and other legal limitations are common to encounter with academic partnerships. Formal relationships (e.g., contracts, MOUs) will be required in some instances. Not all academics have a full understanding of practice level stakeholder needs and this should be considered while developing this partnership. In settings where ‘research’ is not a commonly supported practice, evaluation or developmental evaluation may be more useful ways of characterize the activity [18]. | Use of an academic (university) team to help with planning, designing, evaluation, consultation, training, dissemination etc.  Disseminate the program results through academic outputs (conference presentations, manuscripts, community presentations) |  |  |
| Develop an implementation glossary | Develop and distribute a list of terms describing the innovation, implementation, and stakeholders in the organizational change | When compiling a glossary, reflect as to whether the terms being introduced are essential. | Define key terms, roles, team members, program processes and distribute to the implementation effort. | High level overview either in a meeting, written document, manual, or PowerPoint presentation. |  |
| Identify and prepare champions | Identify and prepare individuals who dedicate themselves to supporting, marketing, and driving through an implementation, overcoming indifference or resistance that the intervention may provoke in an organization | This strategy includes preparing individuals for their role as champions. Champions are primarily internal to the organization. Additional issues raised include the need for guidance regarding:   1. Methods and considerations related to the selection and identification of champions. Social network theory and methods may be useful in this regard. 2. Training and or providing champions support materials. 3. Addressing incentives or disincentives to the champion role. 4. Whether there are needs for champions at different levels of an organization (e.g., clinic, region, national).   Champions are often distinguished from opinion leaders. Opinion leaders may be considered more of an objective third party with relevant expertise. | Internal or external to the organization. Could be administrative leaders, providers, manager, outreach. Help spread the word for the need for the innovation or the effectiveness of the innovation or just get the team excited and on board. | Article must describe someone as a champion, that an individual has gone over an above their role to support the implementation, or retrospectively attributed success thanks to “X” individual. A champion is more of a specific role are champions often know they are champions.  Compare to [inform local opinion leader](#_Inform_local_opinion). *Note: Opinion leaders may be considered more of an objective third party with relevant expertise. Less of an actual “role” but rather just influential individuals you want “on your side” |  |
| Identify early adopters | Identify early adopters at the local site to learn from their experiences with the practice innovation | Early adopters are a good pool for identifying implementation champions. Recruit early adopters to attend stakeholder meetings to present their experiences. Investigating the adoption chasm between early adopters and the early majority has been found to be useful. Different engagement techniques for these two groups are typically needed. For further discussion see Moore [19]. | Specifically seek out providers/admin from pilot/early sites to learn from them. | Code this strategy if manuscript collects experiential data with the purpose of learning from early adopters at provider or organizational level – do not code from participants perspective (i.e., early participant adopters).  Do not code [identify and prepare champion](#_Identify_and_prepare) unless the early adopter is acting as a champion for later sites or involved in stakeholder meetings to drive the implementation. | Focus group with pilot project providers to understand facilitators and barriers to help start-up a new site. |
| Inform local opinion leaders | Inform providers identified by colleagues as opinion leaders or “educationally influential” about the clinical innovation in the hopes that they will influence colleagues to adopt it | The opinions of individuals who refer people to services, or who initiate the connection to services also function in a key opinion role. Keeping opinion leaders informed from pre-implementation through maintenance of the clinical innovation is important. Ensuring that opinion leaders do not serve as implementation obstacles if they are not actively promoting the innovation is also important. | Individual must be outside of the study team. Therefore, an opinion leader cannot also be a champion, supervisor, or facilitator.  Can code for networking purposes to spread the word to *promote* the program. | Compare to [identify and prepare champions.](#_Identify_and_prepare) *Opinion leaders may be considered more of an objective third party with relevant expertise. Less of an actual “role” but rather just influential individuals you want “on your side”. Champions have more of a role. | Inform individual at a local health authority who regularly meets with physicians and/or is respected among physicians and ask them to promote the program to their peers.  Involve the pastor of a church to help with the implementation and recruitment from the pulpit. |
| Involve executive boards | Involve existing governing structures (*e.g.*, boards of directors, medical staff boards of governance) in the implementation effort, including the review of data on implementation processes | Other types of leadership with ‘top-down’ powers may be involved for settings that do not have a governing board. Examples include administrative leadership, clinical leadership, policy makers, and insurance providers or other payment systems. | Governing structures are any organizations that can impact the program. |  |  |
| Model and simulate change | Model or simulate the change that will be implemented prior to implementation | Computer simulations, walkthrough simulation exercises, or modeling the potential overall impact of stakeholder's behavior change may be used. System dynamics modeling is one example of a specific method that may be used [20]. This approach is often more relevant for complex multi-component innovations. | These efforts could involve computer simulations, walk-through simulation exercises, or modeling the overall impact of the program. |  |  |
| Obtain formal commitments | Obtain written commitments from key partners that state what they will do to implement the innovation | Formal commitments should clarify roles, responsibilities, and detail tangible and non-tangible benefits (e.g., community partnerships). Ensure that key roles are supported within the organization (e.g., workload release credit for providing and receiving supervision in a new clinical practice). Formal commitments in no way diminish the importance of informal commitments to a change effort. | Evidence that a contract, memorandum of understanding (MOU) or other document(s) have been signed.  Not at client level (For example, do not code if participants sign a behavioural contract for the program. | See also: [build a coalition](#_Build_a_coalition), [develop resource sharing agreements](#_Develop_resource_sharing) |  |
| Organize clinician implementation team meetings | Develop and support teams of clinicians {the people implementing the program – front line workers) who are implementing the innovation and give them protected time to reflect on the implementation effort, share lessons learned, and support one another’s learning | None | Formal meetings among providers (front-line), admin or anyone implementing the program. Not high-level | Could also be webinars across sites. Must be formal meetings (protected time)  If protected time is not given or lessons learned are shared within or between sites code [create a learning collaboration](#_Create_a_learning) |  |
| Promote network weaving | Identify and build on existing high-quality working relationships and networks within and outside the organization, organizational units, teams, etc. to promote information sharing, collaborative problem-solving, and a shared vision/goal related to implementing the innovation | Individuals functioning as network weavers usually have external links outside of the community to bring in information and ideas. An example would be nurses and doctors who staff hospitals and skilled nursing facilities, and the patients who rotate among these facilities. Networks are somewhat more organic than collaboratives and are often enduring and durable. See: <http://www.networkweaver.com/> | Purposely connect, develop, and seek out partnerships, relationships etc. that contribute to the implementation. | Informal or formal networking.  If specifically recruiting partners in a more formal role, consider [build a coalition](#_Build_a_coalition)  Can code if networking to *optimize* the implementation. | Conduct community engagement events to connect with the broader community “network” to promote the program, build buy-in, gain new insights to improve program, recruitment, infrastructure. |
| Recruit, designate, and train for leadership | Recruit, designate, and train leaders for the change effort | Change efforts require certain types of leaders, and organizations may need to recruit accordingly, rather than assuming that their current personnel can implement the change. Designated change leaders can include an executive sponsor and a day-to-day manager of the effort. Change leaders should consider how to establish effective supervisory lines for clinical practice innovations that are enacted by clinicians when the change leader does not have similar clinical responsibilities. | This can be someone already at the organization (specify their role as implementation facilitator/manager) or someone new (hired an implementation sponsor, manager). | The individual must know they have a leadership role. | Site lead hired to lead trained program staff at each site, host monthly meetings. |
| Use advisory boards and workgroups | Create and engage a formal group of multiple kinds of stakeholders to provide input and advice on implementation efforts and to elicit recommendations for improvements | Consider how group composition (or heterogeneity) impacts stakeholder participation and take active steps to reduce response bias. For example, inclusion of supervisors and supervisees in these groups can be problematic and it may be a desirable strategy to ensure that these situations are avoided due to the power difference in the relationship. Supervisees, for example, may feel pressure to report positively to put a good face on for the supervisors, and supervisors may feel pressure to deny having any problems with implementation to save face. It can be useful to distinguish between internal stakeholders and representatives (in a participatory approach to maintain buy-in and relevance) versus external experts and advisors. Similarly, depending on the input or oversight need, the workgroup composition may include multiple-level or multi-disciplinary stakeholders. | Seek advice from a formal group of key stakeholders. Must be purposeful to collect feedback and advice on implementation.  Can be a one-time meeting or ongoing, before implementation or during implementation. | Must be a formal group that meets. | Participant advisory board, stakeholder advisory board |
| Use an implementation advisor | Seek guidance from experts in implementation | This could include consultation with outside experts such as university-affiliated faculty members, or hiring quality improvement experts or implementation professionals. | This might include outside experts – professors, quality improvement experts, implementation professionals. | Advice is at the broad implementation level, organizational level.  Typically, high level program developers, researchers who need advice on broad implementation questions.  If advice is given at the level of the providers needing help to implement sessions/the program, consider [Provide ongoing consultation](#_Provide_ongoing_consultation) |  |
| Visit other sites | Visit sites where a similar implementation effort has been considered successful | Clarifying the goals of the site visit prior to making the visit is particularly useful. Comparing and contrasting the features of one’s own site with the comparison site in preparation for the visit may better inform the visit objectives. Clarifying goals, in part includes developing a plan for using the information upon returning to your setting. Identify adaptations made in implementing the innovation and any perceived impact on the effectiveness of the innovation/practice change. It is important to document facilitators and lessons learned. Much can be learned from visiting sites that have a strong track record for successfully implementing a wide variety of other innovations/practice changes. Consulting with sites where implementation has stalled or failed can also provide useful information. Sites also benefit from sharing implementation planning and execution notes virtually (i.e., information exchange is not limited to physical visits). | Site visits. | Visit a site to learn from it (positive or negative lessons), about implementation.  If completing a site visit to audit sites, consider coding [Audit and provide feedback](#_Audit_and_provide)  If sharing lessons learned is completed consider coding [capture and share local knowledge](#_Capture_and_share) |  |
| Increase demand | Attempt to influence the market for the clinical innovation to increase competition intensity and to increase the maturity of the market for the clinical innovation | One way of increasing demand is to educate patients about the clinical innovation so that they demand it from their providers (e.g., what pharmaceutical companies do). | Purposely attempt to increase the demand for the DPP – raise awareness among patients, providers, health authorities, government to want/ask to provide the program. | Code this strategy if using community recruitment events to increase demand.  If using mass media to increase demand, code [use mass media.](#_Use_mass_media) |  |
| Intervene with patients/consumers to enhance uptake and adherence | Develop strategies with patients to encourage and problem solve around adherence | This includes patient/consumer reminders and financial incentives to attend appointments.  Feedback regarding patient/consumers' understanding and use of the treatment is also important to collect. | Providing incentives is an example of an intervention to encourage adherence, this includes recruitment incentives.  You can also code this strategy if participants provide feedback on how to improve adherence and uptake (e.g., have after work hour classes, send reminder emails before appointments) | If including participants for general feedback code: [Obtain and use patients/consumers and family feedback](#_Obtain_and_use)  If including participants to make decisions or get their opinion in the implementation planning, consider: [Involve patients/consumers and family members](#_Involve_patients/consumers_and)  If incentive is at the provider level, consider [Alter incentive/allowance structures](#_Alter_incentive/allowance_structure) | Hold interviews, collect survey data and/or focus groups with participants for (prospective or retrospective) feedback on the uptake (enrolment) and adherence (attendance) |
| Involve patients/consumers and family members | Engage or include participants/consumers and families in the implementation effort | Feedback from stakeholders can be obtained *at any stage of the implementation process* depending on the needs and goals of project. Involving stakeholders in the pre-implementation phase for many innovations is advantageous. Training in the innovation, and relevant advocacy, may also be included in stakeholder involvement. Informal caregivers such as neighbors, friends, and other key sources of support may also be prudent to include. | Clients need to be included in the *implementation effort* e.g., development, planning, evaluation. This can be at any phase; development, training, recruitment, dissemination.  Code for events used to involve, engage or consult participant stakeholders on the implementation effort [involve in decision-making]. This strategy is different than obtaining feedback. If specifically including pathways for participants to provide *(continuous) feedback* during or post-program (participants), code [Obtain and use patients/consumers and family feedback](#_Obtain_and_use) instead. | This can be at the program level. NOTE: if specifically obtaining feedback on uptake (enrolment) or adherence (attendance) code [Intervene with patients/consumers to enhance uptake and adherence](#_Intervene_with_patients/consumers)  If specifically obtaining feedback (from interviews, surveys, etc.) from participants after program participation, code [Obtain and use patients/consumers and family feedback](#_Obtain_and_use) | Involve participants in the planning phase, evaluation or meetings about the implementation to understand how to meet their needs. |
| Prepare patients/consumers to be active participants | Prepare patients/consumers to be active in their care, to ask questions, and specifically to inquire about care guidelines, the evidence behind clinical decisions, or about available evidence-supported treatments | Preparing consumers to inquire about specific practices can involve asking questions, and educating patients/consumers about the existence of treatments supported by evidence, as well as explicitly inviting them into the process of treatment decision-making. | Educational outreach to the community or to program participants that specifically aims to empower them to take control over their prediabetes and their care decisions. If there are educational outreach events in the community to target participants to get to know the program. | Most often this is a sort of “primer” before the program starts. Does not need to be part of the program. Can code this strategy for participant level outreach events e.g., community recruitment events that target educating the community about prediabetes or a specific program.  Can be part of the program, but then it must be a clear focus of the session/consultation/program, such as a pre-program intake session. |  |
| Use mass media | Use media to reach large numbers of people to spread the word about the clinical innovation | Mass media may include television, newspapers, magazines, radio, electronic social media, listservs, mass email campaigns, mass mailings, and robocalls as methods for spreading information. Targets of these media campaigns may be clinicians, potential consumers of the innovation, or their associates. Other commonly used terms include marketing or social marketing. | newsletters, social media, public service announcements, magazines, commercials, podcasts… to spread information about the program. | Code if media used for recruitment, dissemination, awareness or any aspect of implementation.  If specific target of mass media is to increase the number of sites delivering program/recruit new sites, OR to prompt participants to talk to their provider about the program consider [increase demand.](#_Increase_demand) |  |
| Centralize technical assistance | Develop and use a centralized system to deliver technical assistance focused on implementation issues | This could be the designation of a lead technical assistance organization (could also be responsible for training). The lead technical assistance entity can develop other mechanisms (e.g., call-in lines or websites) in order to share information on how to best implement the clinical innovation. | Could be its’ own organization or part of the implementation initiative. Can be a person or a team. Can also be in the form of support websites, call-centers to share information on how best to implement the program. Especially to multiple sites. | Compile all information and project assistance in one place. In a multi-site program, all sites have access to this one central person/group/call center/website. In a single-site program first examine [provide local technical assistance](#_Provide_local_technical) to see if that code fits. |  |
| Facilitation | A process of interactive problem solving and support that occurs in a context of a recognized need for improvement and a supportive interpersonal relationship | Facilitation can be internal or external to a system. This interactive support process can include a combination of any implementation strategies, and typically bundles multiple strategies as needed. | Coaching, education and/or feedback general support from facilitator.  This strategy is at the organizational level, NOT at client level. E.g., facilitation of the providers, admin, staff and NOT facilitation of the clients. | If providing assistance, specifically organizational level assistance, at the local level or multi-site level, consider code [provide local technical assistance](#_Provide_local_technical) or [centralize technical assistance](#_Centralize_technical_assistance)  If goal is to facilitate/coach/support providers in a supervisory role, consider code [provide clinical supervision](#_Provide_clinical_supervision) |  |
| Provide clinical supervision | Provide clinicians with ongoing supervision focusing on the innovation. Provide training for clinical supervisors who will supervise clinicians who provide the innovation | Clearly defining the role of supervision and providing ongoing resources to ensure that it occurs can be important. Supervisor training often needs to include specific training in how to supervise the innovation.  See Nadeem et al. [13] for a discussion of the distinction between consultation and supervision. | Supervisor can be tasked with overseeing providers’ performance, provide support, guidance, manage relationship between providers and larger organization, provide study updates to providers | Support is mostly for provider-level, but could also be for support staff (e.g., administrative staff)  If someone is providing support/advice/coaching specific to certain skills, consider coding [Provide ongoing consultation](#_Provide_ongoing_consultation)  If there is a specific training for supervisors or other leaders in the implementation effort, review code: [Recruit, designate, and train for leadership](#_Recruit,_designate,_and) | Site lead is responsible for overseeing trained staff. Trained staff understand they can go to the site lead for support. |
| Provide local technical assistance | Develop and use a system to deliver technical assistance focused on implementation issues using local personnel | Local technical assistants can be connected with a broader or centralized network of technical assistants. Technical assistance for both the clinical innovation and the implementation processes may be important. For example, the VA aims to have mental health Evidence-Based Psychotherapy coordinators, Military Sexual Trauma coordinators, and OEF/OIF/OND coordinators in each facility who can provide technical assistance to other local clinicians for relevant initiatives. | Can be a person or a team local to the implementation site to share information on how to best implement the program. | Local support using personnel. If multi-site, code this strategy if someone local is designated for support. If support systems are put in place that do not include personnel (e.g., website or online training program) consider [centralize technical assistance](#_Centralize_technical_assistance). If support is centralized, consider code [centralize technical assistance](#_Centralize_technical_assistance).  If the goal is to just provide supervision of providers, code [provide clinical supervision](#_Provide_clinical_supervision) | The supervisor cannot also be the person who provides technical assistance. The person who provides technical assistance would be who the site lead/ supervisor contacts with questions. |
| Create new clinical teams | Change who serves on the clinical team, adding different disciplines and different skills to make it more likely that the clinical innovation is delivered (or is more successfully delivered) | none | Adding a new role to the team. For example, hire a dietician to provide nutrition lessons for the diabetes prevention program or a kinesiologist for exercise sessions. | Code if a NEW grouping of health professionals or a new type of health professional is a result of the program implementation. | Perhaps typically just the physician completes diabetes consultations but now there is also a physiotherapist, dietician, psychologist, administrator, health promotor, volunteer etc. This would be a “new” team. |
| Develop resource sharing agreements | Develop partnerships with organizations that have resources needed to implement the innovation | For example, this could involve data sharing agreements, agreements to share necessary equipment (e.g., telemedicine equipment), or sharing the cost of bringing in experts who provide training and consultation. Resource sharing agreements could involve formal memorandums of understanding (MOUs), or be much more informal in nature. | Resources are broad, for example, laboratories, new program sites (space for exercise, cooking demonstrations, meeting rooms). Partner with a lab to help recruitment. | Do not code if resources are readily available to general population e.g., using published educational documents, routine lab work. | Partner with a local laboratory to flag individuals with a HbA1c within the prediabetes range. |
| Facilitate relay of clinical data to providers | Provide **as close to real-time data** as possible about key measures of process/outcomes using integrated modes/channels of communication in a way that promotes use of the targeted innovation | For recommendations regarding how to introduce innovation or change of any kind into existing work flows, please see May [30]. | Provide outcome data (weight loss, activity level) or process data (referral numbers, attendance) to providers. | Must have a documented process to relay the information. Feedback goes directly to those directly facilitating the program.  If the data that is relayed is specific to quality monitoring, consider coding [Develop and implement tools for quality monitoring](#_Develop_and_implement) and [Develop and organize quality monitoring systems](#_Develop_and_organize) |  |
| Remind clinicians | Develop reminder systems designed to help clinicians to recall information and/or prompt them to use the clinical innovation | Reminders could be patient or encounter-specific, provided verbally, on paper, or electronically. Computer-aided decision support, and drug dosages are included in this strategy. Reminders may be delivered at various time points (prior to service, during service, or following service delivery). | Reminder could be on paper, verbal or on computer. Reminder could be used for providers during a session or other reminders e.g., recruitment processes. | Does not have to be clinician specific but reminder to anyone on program team, to help them remember the innovation. | Checklists for use during a session can include written reminders for providers. |
| Revise professional roles | Shift and revise roles among professionals who provide care, and redesign job characteristics | Revising professional roles includes the expansion of roles to cover provision of the clinical innovation and the elimination of service barriers to care, including personnel policies. | This may mean expanding current roles to cover the program or adding new assignments (completing paperwork, data entry, collection, recruitment etc.) to current roles. | Code if a departure from the normal staff duties. | If fitness facility staff can now also counsel on diet.  Ask admin staff at fitness facility to enter data into computer. |
| Conduct educational meetings | Hold meetings targeted toward different stakeholder groups (*e.g.*, providers, administrators, other organizational stakeholders, and community, patient/consumer, and family stakeholders) to teach them about the clinical innovation | The content of the education may include information regarding what to expect as implementation moves forward. It is useful to ensure that meeting attendees are relatively homogeneous so that the education can be targeted toward the stakeholder group’s needs. For example, some educational meetings may inform the stakeholder group about the clinical innovation in a way intended to increase demand, while others may preview the clinical innovation for providers and administrators. It is often useful to have recordings or other materials from the educational meetings available to those who cannot attend the meetings (e.g., those covering patient care at the time of the meeting, new hires subsequent to the meeting). | This can be formal events (e.g., community presentation) to teach the community about the initiative, or to gain support/ buy-in within an organization (e.g., start-up meeting).  Targeted training for different members of the team. | Code for introductory or first encounter educational events. For ongoing training or educational events see: [Conduct ongoing training](#_Conduct_ongoing_training)  If the educational meeting is an “outreach” opportunity specifically at the practice setting, and at provider level, do not code and consider [conduct educational outreach visits](#_Conduct_educational_outreach) instead.  If at participant level, consider [Prepare patients/consumers to be active participants](#_Prepare_patients/consumers_to) If the outreach/education is specific to raise awareness about the program and empower participants to make decisions about their care, | Have a member of the research team conduct a start-up meeting to raise awareness and teach providers about the innovation. |
| Conduct educational outreach visits | Have a trained person meet with providers in their practice settings to educate providers about the clinical innovation with the intent of changing the provider’s practice | Visits to the site may be in-person or virtually via the Internet. Some initiatives may require regular educational outreach as part of maintaining the innovation/practice change. Academic detailing is another commonly used term, although academic detailing typically involves many additional discrete implementation strategies (e.g., conduct ongoing training, modeling, developing and distributing educational materials; [21, 22]). | “Academic detailing”, site recruitment opportunities, physician referral recruitment meetings. A form of dissemination of the program. | Must be a specific meeting with an intent to change behaviour (individual – physician or organizational – site) to **adopt** the program.  If the education is general to raise awareness about the program, consider [conduct educational meetings](#_Conduct_educational_meetings) instead.  **Do not code if outreach events are for** **participant recruitment**. If the outreach/education is specific to raise awareness about the program and empower participants to make decisions about their care, consider [Prepare patients/consumers to be active participants](#_Prepare_patients/consumers_to) | Lunch and learn for physicians to get them aware of the program and with the intent of receiving referrals from them to the DPP. OR lunch and learn with potential new sites to advocate that they want to implement the program. |
| Conduct ongoing training | Plan for and conduct training in the clinical innovation in an ongoing way | This can include follow-up training, advanced training, booster training, purposefully spaced training, training to competence, integration of off the- job and on-the-job training, structured supervision, the introduction of concepts in a specific sequence to ensure mastery, and trainings based on the level of clinician knowledge. Ongoing training efforts need to reach across shifts and accommodate staff turnover, as well as rotating staff (e.g., residents). Trainings can be in-person, on the web, or technology-assisted (e.g., simulation lab training), and may focus on individuals or involve groups. When planning for ongoing training, it is important to describe the training components, including the timing and frequency of trainings. Issues related to the dynamics of training can be found in the strategy, [make training dynamic](#_Make_training_dynamic). | Formal follow-up training (booster, advanced training, purposefully spaced – part two of the training 2 months later, train to competence… etc.) | Code formal trainings only. Do not code ongoing informal, or one-on-one support.  If the description of the training describes multiple modes (e.g., role-play, discussion, online portal, didactic etc.) also code [make training dynamic](#_Make_training_dynamic).  This can be training for the provider level or implementation process level (e.g., admin, recruitment)  If help/ ”training” is more “as needed” or providers are told to seek advice from specific “experts” for help with skills, consider [provide ongoing consultation](#_Provide_ongoing_consultation)  If more informal ongoing feedback comes from an individual from a supervisory level. Consider [provide clinical supervision](#_Provide_clinical_supervision) | Coaches completed an initial training and then met again in 6 months for a booster training. |
| Create a learning collaborative | Facilitate the formation of groups of providers or provider organizations and foster a collaborative learning environment to improve implementation of the clinical innovation | There are several approaches to this in the literature including peer consultation networks, online communities of practice, quality circles, and learning collaboratives. Groups may meet in person or interact using a wide variety of media. The inclusion of a quality manager within the collaborative may be useful. Positive deviance approaches use “discovery and action dialogue” among peers to promote collaborative learning [23–25].  Resources specific to learning collaboratives include:  The Health Resources and Services Administration (HRSA) [26]  The Institute for Healthcare Improvement (IHI) [27, 28]  Key terms for searching literature specific to collaborative learning include: learning community, learning network, and community of practice. | May be called peer consultation networks, community of practice (COP), quality circles. This is at the level of those at the front-line (directly interacting with participants) | To code, must be a formal groups with a focus on collaborative learning  If the groups are specific to those implementing the program, consider also coding:  [Organize clinician implementation team meetings](#_Organize_clinician_implementation) | Hold a monthly meeting for front-line staff to discuss successes, barriers, challenging scenarios and lessons learned. |
| Develop educational materials | Develop and format manuals, toolkits, and other supporting materials in ways that make it easier for stakeholders (anyone supporting the program – physician, pharmacist, church leaders) to learn about the innovation and for clinicians (providers) to learn how to deliver the clinical innovation (DPP) | Create eye-catching, easy-to-use educational documents. Distill complex information into easier-to-learn components. Consider teaching skills modularly. Use different forms of media, and target messages for different audiences. Educational materials should reflect principles of adult learning theory. Assessment of current, available technology infrastructure to accommodate educational media (e.g., firewalls, old hardware, old software) is merited. Consider how the educational materials will be used over time. For example, will the educational materials’ primary use be to train new or rotating staff; or to refresh staff knowledge; or to be incorporated into existing supervision, competency, and performance review structures. Educational materials may be refined through the use of formative evaluation feedback.  Relevant suggestions are provided via the REP framework, under its ‘packaging’ domain [7]. Further support related to developing educational materials can be found on the Training Within Industry Service website [29]. | This strategy is not for materials for clients (e.g., program workbook, program flyer).  This strategy is at the organizational level. Can be materials for providers (manual, scripts, PowerPoint slides) or for other stakeholders (e.g., physician/ pharmacist/ church leadership buy-in). Help educate recruiters. Can also include materials on processes (e.g., overview of workflow, standard operating procedures). | Do not code if materials are developed for participants.  Any material that was developed to aid with supporting the community organization to learn about the program or about diabetes (e.g., training manual). This can also include materials online. Do not code for developing program level documents e.g., program workbook for participants.  If materials are distributed, also code: [Distribute educational materials](#_Distribute_educational_materials) | training manual, online training, letter to physicians describing the program. |
| Distribute educational materials | Distribute educational materials (including guidelines, manuals, and toolkits) in person, by mail, and/or electronically | none | For organizational level.  Do not code if distributing materials to client (e.g., program workbook) | Do not code if educational materials are distributed to clients.  Only code for formally developed materials.  If materials were developed for the implementation also code: [Develop educational materials](#_Develop_educational_materials) | Each provider received a training manual. |
| Make training dynamic | Vary the information delivery methods to cater to different learning styles and work contexts, and shape the training in the innovation to be interactive | Making training dynamic includes efforts to divide material into small time intervals, the use of small group breakouts, audience response systems, and other measures, such as having learners try new skills between training sessions. Interactive components of training can be very dynamic with participants actively contributing to the training content, engaging in problem solving, and identifying solutions that can be tested. | Use of break-out groups, role-play, quizzes, power-point, mock sessions. Not just lecture-style education. There must be evidence of more than one delivery method during a training.  Training is at the level of the provider (who implements the DPP program). Do not code at participant level. | If a formal training, code: [Conduct educational meetings](#_Conduct_educational_meetings). If an ongoing training, code: [conduct ongoing training](#_Conduct_ongoing_training) |  |
| Provide ongoing consultation | Provide ongoing consultation with one or more experts in the strategies used to support implementing the innovation | Ongoing consultations could include in-person or distance consultation and feedback on taped clinical encounters. Consultations are tailored to the clinician’s actual practice, thus, differentiating a consultation from ongoing trainings. Feedback may be from a consultant external to the organization, which distinguishes consultation from clinical supervision. Some practice changes can involve a recertification process, thus, involving consultation ensures adequate fidelity. Consultation may also be necessary for non-clinical staff such as administrators and those responsible for billing, constructing feedback systems, or other staff with duties that impact the implementation process. | Consultation at the level of implementation, or at the level of providers. Should be on an add-needed basis or one-on-one instead of a group and from an expert. | If consultation occurs at regular interval and is for all providers, consider [conduct ongoing training](#_Conduct_ongoing_training).  If consultation to support the individuals facilitating program sessions and comes from someone within the organization, consider code: [provide clinical supervision](#_Provide_clinical_supervision)  If advice is needed at the high level broad overview of the entire implementation project in general, consider [Use an implementation advisor](#_Use_an_implementation) | If a frontline staff is struggling with motivational interviewing, they can consult with an expert of motivational interviewing for additional support. |
| Shadow other experts | Provide ways for key individuals to directly observe experienced people engage with or use the targeted practice change/innovation | While shadowing traditionally has involved in-person observation, creative use of technology may provide additional opportunities for individuals to observe and learn from those experienced in the innovation. | Must describe shadowing in the article | Code if this is part of training or onboarding protocols. | New coaches shadow experienced coaches prior to having their first participant. |
| Use train-the-trainer strategies | Train designated clinicians or organizations to train others in the clinical innovation | Restrictions regarding who can serve as a trainer are idiosyncratic to the innovation or practice change, for example, some innovations may require that supervisors have specific levels of education, training, or experience, and such restrictions should be explored in the planning phase. Train-the-trainer strategies may also apply to those responsible for administrative procedures, and who are part of implementing the innovation. | If training was led by a previously trained individual, or article mentioned that trained individuals can then train new providers to deliver the organization. | If an organization conducts the training, consider code: [work with educational institutions](#_Work_with_educational) | New coaches are trained by coaches with over 1-year of experience. |
| Work with educational institutions | Encourage educational institutions to train clinicians in the innovation | This strategy fits well with innovations requiring clinical training and other skills where training expertise is more likely to be housed in educational institutions. | Not just for training but also for supporting or disseminating implementation | If a program is partnered with an academic institution  consider: [develop academic partnerships](#_Develop_academic_partnerships) |  |
| Assess for readiness and identify barriers and facilitators | Assess various *aspects of an organization* to determine its degree of readiness to implement, barriers that may impede implementation, and strengths that can be used in the implementation effort | Readiness assessments may focus on agency finances, staffing levels, and other material or logistical resources needed, or available, to support the implementation effort. Further this assessment may also focus on leadership support, the organizational priority for change, and the presence of successful experience with quality improvement techniques and change management. Additional aspects for assessment may include other services provided, as well as community support, stakeholder attitudes, and beliefs and perceptions of evidence for the innovation or change. Rationale for current practices, organizational climate and culture, structure, decision-making styles, and the perceived needs of frontline stakeholders to implement the change or innovation (consider adaptation needs and limits) are also important aspects to consider in this assessment. Readiness assessments can be used to vet, eliminate, or prioritize implementation sites. More so, the assessment can help make internal decisions about whether to go ahead with an implementation initiative. Some barriers can be difficult to observe prior to implementation. Specific measures have been created to assess readiness for change, which may be useful (e.g., [1–3]). | Assessment may focus on finances, other services provided, community support, clinician attitudes and beliefs, organizational climate and culture, structure, and decision-making styles. OR use of specific site readiness assessments. | Focused on context, front-line workers, and other personnel; barriers to implementation, Includes formal (e.g. baseline focus groups) and informal methods for gathering this information. | Site start up meeting to discuss barriers, discuss strategies.  Discuss necessary changes to infrastructure to accommodate the program. |
| Audit and provide feedback | Collect and summarize clinical performance data (process variables, outcome variables, observation) over a specified time period and give it to clinicians and administrators to monitor, evaluate, and modify provider behavior | The information may be obtained from a variety of sources, including medical records, computerized databases, observation, or feedback from patients. Performance evaluations may also be considered as audit and feedback data if the evaluation included specific information on clinical performance. Feedback summaries may include recommendations. Feedback may be displayed publicly, and often involves comparisons to peers or to local, state, national, or international norms. Feedback may be designed to guide a clinician in improving fidelity. It should also be noted that audit and feedback data can be helpful in promoting the continuation of intended behavior. Performance data may include process variables, outcomes, or fidelity measures. Feedback can include mandatory performance measures, which are related to benchmarks from the literature or normative data within an organization or industry. | Feedback can be in the form of presentations, report, consultation.  Code this strategy if manuscript mentioned “performance evaluation” | Do not code if there is an audit and no feedback to clinicians/administrators.  Collecting data and presenting in a manuscript is not sufficient for audit and feedback. |  |
| Conduct cyclical small tests of change | Implement changes in a cyclical fashion using small tests of change before taking changes system-wide. Tests of change benefit from systematic measurement, and results of the tests of change are studied for insights on how to do better. This process continues serially over time, and refinement is added with each cycle | Two common small tests of change cycling strategies are “Plan-Do-Study-Act” from Deming’s quality management work [4], and six sigma’s Define- Measure- Analyze-Improve-Control sequence [5]. | “Plan-Study-Do-Act” is an example of change cycling strategies in the literature. Same with Define-Measure-Analyze-Improve-Control Sequence. |  | Pilot a change at one site before implementing it at all sites. |
| Conduct local needs assessment | Collect and analyze data related to the need for the innovation | This assessment could be focused on:   - Outcomes of usual care - Process of care - Description of usual care and its distance from evidence-based care (e.g., gaps in care) - Opinions from stakeholders (including patients) on (a) barriers and facilitators to the desired outcome (e.g., recovery from mental illness), (b) the need for any innovation (i.e., tension for change), (c) the need for a specific innovation, or (d) the special considerations for delivering the innovation in the local context.   Common needs assessment methods include surveys, focus groups, key informant interviews, direct observation, and data mining of administrative records utilized to identify target populations, as well as identify baseline care process and outcome clinical care data. If the change involves multiple sites or facilities, then it is necessary to examine practice variation across facilities, and outline strategies for the needs assessment to support a standardized approach across sites. Collecting data from a random sample of stakeholders may be necessary to reduce response bias and decrease chances that the level of need is not over or under-estimated. | Could be focused on collecting data (survey, focus group, interviews, document/data analysis) from various stakeholders, participants, etc. for:   1. Data they want collected as part of implementation or program evaluation 2. Date demonstrating high levels of diabetes and/or prediabetes in an area 3. Need for a diabetes prevention program in a certain community 4. Physicians/participants/health authority describing the need for a DPP 5. Barriers and facilitators to reducing diabetes risk 6. special considerations for delivering the DPP in the local context (site specific needs) | Often before implementation begins.  Consider coding these strategies if the needs assessment leads to modifications to the program [(promote adaptability](#_Promote_adaptability)) or the implementation strategies ([tailor strategies](#_Tailor_strategies)) |  |
| Develop a formal implementation blueprint | Develop a formal implementation blueprint that includes all goals and strategies. The blueprint should include the following: 1) aim/purpose of the implementation; 2) scope of the change (e.g., what organizational units are affected); 3) timeframe and milestones; and 4) appropriate performance/progress measures. Use and update this plan to guide the implementation effort over time | The implementation blueprint or manual may be informed by one or more theories or conceptual frameworks and/or data from pre-implementation needs assessments. This blueprint can also provide a useful historical record of the implementation process, as well as provide a mechanism to track changes over time. The implementation blueprint is often useful to ensure feedback is received from prospective frontline users of the blueprint prior to implementation.  Consider coordinating this strategy with the development of a fidelity monitoring tool.  Issues to consider separately, especially for research purposes:   - Number and type of implementation strategies - Organizational levels involved—this can vary by type of intervention. It may be possible to do some interventions at the lowest level. Others may require top management. - Pre-implementation assessments would be separate step   Other examples of how to create an implementation blueprint include the CDC's Replicating Effective Programs [6, 7].  Examples of projects using a blueprint include:   - HI-TIDES [8] - Depression in a substance abusing population [9]   Mental health services in federally qualified health centers [10] | Plan can include staffing, funding, monitoring. Use of theory or multiple theory. Update the plan overtime.  To code this strategy, there must be written evidence that there were products from the planning discussions e.g., Gantt charts, written planning document, blueprint, protocol. |  |  |
| Develop and implement tools for quality monitoring | Develop, test, and introduce into quality-monitoring systems the right input—the appropriate language, documents, protocols, algorithms, standards, and measures (of processes, patient/consumer outcomes, and implementation outcomes) that are often specific to the innovation being implemented | These tools should be flexible enough to reflect fidelity, even after adaptations to the setting or client. Performance sites can benefit when these tools are available locally, particularly to help clinicians develop a sense of ownership for the change process. Quality monitoring tools can be coordinated with other strategies to encourage or reward performance that is in alignment with the clinical innovation. See Krein et al. [11] for an example of this process. | This strategy focuses on data collection tools. Quality monitoring can be related to implementation outcomes (fidelity, adherence) or program outcomes (adherence, recruitment reports). | Code if data collection tools specific to quality monitoring.  If an article described they did “quality monitoring” without describing WHAT they monitored or HOW, do not code this strategy, instead code [develop and organize quality monitoring systems](#_Develop_and_organize) | Implement fidelity checklists that frontline staff must complete during the session. |
| Develop and organize quality monitoring systems | Develop and organize systems and procedures that monitor clinical processes and/or outcomes for the purpose of quality assurance and improvement | This includes developing systems for monitoring through peer reviews, collecting data from patients and consumers, clinicians, and supervisors, and using administrative and electronic record data. This category of strategies also includes the design of disease-specific clinical registries, where clinical information and tools (graphical representations, real-time report cards, comparisons with benchmarks, etc.) are available to care team members. These systems may inform audit and feedback strategies. Some intensive fidelity monitoring activities (e.g., psychotherapy recordings) are more practical at random, but not infrequent, intervals. | This includes developing systems for monitoring could be at the administrative level, electronic data (database), or disease-specific clinical registries. The monitoring should be continuous. | This strategy focuses on the infrastructure or system to collect the data. Development and/or modifications to systems.  These systems may inform [audit and feedback](#_Audit_and_provide) strategies and may use specific [tools for quality monitoring](#_Develop_and_implement). Code accordingly.  If an article described they did “quality monitoring” without describing WHAT they monitored or how, code this strategy but do not code [develop and implement tools for quality monitoring.](#_Develop_and_implement) | All sessions are audio recorded for fidelity purposes.  Every three month a supervisor watches a coach implement the program for fidelity purposes (continuous data collection system) |
| Obtain and use patients/consumers and family feedback | Develop strategies to increase patient/consumer and family feedback on the implementation effort | This can continue throughout the implementation effort. Strategies could include complaint forms, or methods, which funnel feedback to change managers or advisory boards. Consider whether anonymous feedback formats are appropriate. | Specific methods to collect participant feedback – survey, complaint form, interviews, focus groups  Do not code this strategy for feedback BEFORE program implementation  Client feedback | Strategy is at client level.  If collecting feedback specifically on retention/adherence, consider coding: [intervene with patients/consumers to enhance uptake and adherence](#_Intervene_with_patients/consumers)  If obtaining feedback prior to implementation or during design process, consider coding [conduct local needs assessment](#_Conduct_local_needs) if feedback is at the program level. If feedback is about understanding if there is a need for a program or understanding if diabetes is important to participants, consider [Conduct local consensus discussions](#_Conduct_local_consensus) | Client interviews to understand participant feedback on the program. |
| Purposely reexamine the implementation | Monitor progress and adjust clinical practices and implementation strategies to continuously improve the quality of care | It is beneficial to use a concrete schedule for monitoring rather than ‘as needed.’ Time-sensitive benchmarks for determining when adjustments are needed have also been found to be useful. | Formal discussions on implementation successes and challenges to make changes to the process/plan as needed. | Similar to quality monitoring or audit and feedback strategies, however, broader, may not be data-driven (compared to quality monitoring which requires collection and review of data) – a change may result after a formal or informal discussion. Modifications can be implemented anywhere. | Often in the form of meetings. Higher level progress and practice level that might not be caught by quality monitoring. |
| Stage implementation scale up | Phase implementation efforts by starting with small pilots or demonstration projects and gradually move to a system wide rollout | This involves an iterative process that often results in adaptations. Strategies for integrating pilot feedback into the scale-up or spread process should be established in advanced. Depending on the innovation, piloting may also involve phasing in elements or components of the practice change. Many innovations involve more than one service (e.g., inpatient and outpatient; primary care and specialty care), and the scaling-up or spread process may have different needs to address the interactions among services (e.g. needs related to ensure continuity of care while connecting services). For more details see the Institute for Healthcare Improvement’s white paper, which describes a framework for spread [12]. | Need evidence in the manuscript that the program was/is being built on smaller pilot projects to larger projects. | Code this if prior research included a pilot or feasibility study (or in general this program is building from prior research)  Only code if a larger study built off of a smaller study. If you are reading a pilot study, do not code this strategy because we don't know if a larger study was informed by it. | NHS program started with 4 demonstrator locations. |
| Access new funding | Access new or existing money to facilitate the implementation | Accessing new funding sources could involve new uses of existing money, accessing block grants, shifting funding from one program to another, cost-sharing, passing new taxes, raising private funds, or applying for grants. These monies may be used to fund the delivery of a clinical innovation, or to support other time limited actions needed for initial implementation, such as to purchase material or logistical support, training, and consultations. | This could involve new uses of existing money; applying or winning new grants; shifting funding from one program to another; cost sharing; passing new taxes; or raising private funds; | Code this for mention of where funding came from for the project. |  |
| Alter incentive/allowance structures | Work to incentivize the adoption and implementation of the clinical innovation (DPP)  **Work to incentivize **providers or an organization** to adopt and implement the DPP | Incentives may be based on the performance of individual clinicians or larger performance units at the organizational level. The incentive could be in the form of an increased rate of pay to cover the incremental costs associated with implementing the clinical innovation. The incentive could be through loan reduction or forgiveness to clinicians to learn an innovation. This category of financial strategies also includes the elimination of any perverse incentives that become a barrier to receiving appropriate care. An incentive suggests the payment is tied to performing a clinical action or improving outcomes. An allowance suggests that the clinician or organization is not required to perform the clinical action or meet the performance standard. | Not at participant level.  Incentive at staff level. | At organizational level.  Do not code if incentives are at the participant level or recruitment level. Rather review: [Intervene with patients/consumers to enhance uptake and adherence](#_Intervene_with_patients/consumers)  Incentives are generally financial incentives. Incentives or benefits that result from a mutually beneficial partnership are not be sufficient for this code. Review [Develop resource sharing agreements](#_Develop_resource_sharing) |  |
| Alter patient/consumer fees | Create fee structures where patients/consumers pay less for preferred treatments (the clinical innovation - DPP) and more for less-preferred treatments | none | This strategy makes the program easier for clients to participate (strategy is at client level) | Client level. Code if the program is free or a reduced fee. | Free program |
| Develop disincentives | Provide financial disincentives for failure to implement or use the clinical innovations | In addition to direct financial disincentives, this strategy could include tying promotion decisions to the use of certain innovations. | Penalize providers financially for failure to implement or use the clinical innovation. | Organizational level. | If a site onboards to participate in delivering the program but never has a single participant and so the organization fines them. |
| Fund and contract for the clinical innovation | Governments and other payers of services issue requests for proposals to deliver the innovation, use contracting processes to motivate providers to deliver the clinical innovation, and develop new funding formulas that make it more likely that providers will deliver the innovation | none | If an organization puts out requests for other organizations to enrol in the DPP and start a new site. Request comes from organization.  If the implementation team/organization advocates to and is successful in getting the government or insurance providers to provide coverage for the innovation (DPP) so that the program is available at a reduced rate for participants. | Scale-up level within an organization OR specific money from governments or insurance providers to support the innovation.  Code this strategy if a program is covered under insurance policies (participants can make claims to their insurance provider for participation). | The United States National Diabetes Prevention program has insurance coverage |
| Make billing easier | Make it easier to bill for the clinical innovation | Making billing easier might involve requiring less documentation, ‘block’ funding for delivering the innovation, and creating new billing codes for the innovation. Developing progress note templates to facilitate documentation of the clinical innovation can also decrease the burden for obtaining payment. | Less documentation, set of money for the overall implementation (block funding) or new billing codes for the program | Billing at organizational level. |  |
| Place innovation on fee for service lists/formularies | Work to place the clinical innovation on lists of actions for which providers can be reimbursed (e.g., a drug is placed on a formulary, a procedure is now reimbursable) | none | If diabetes prevention program is placed on a list that providers get reimbursed for. | At organizational level. |  |
| Use capitated payments | Pay providers or care systems a set amount per patient/consumer for delivering clinical care | This is an implementation strategy to the degree that it frees the clinician to provide services that they may have been disincentivized to provide under a fee-for-service structure. This may be helpful to motivate clinicians to use certain clinical innovations. These changes often come about as part of policy changes that alter fee structures, alter coverage, or add items to reimbursement formularies. | This is an implementation strategy to the degree that it frees the clinician to provide services that they may have been disincented to provide under a fee-for-service structure. This may be helpful to motivate clinicians to use certain clinical innovations. | Payments scheme at organizational level. | Payment scheme to motivate clinician to use the program. |
| Use other payment schemes | Introduce payment approaches (in a catch-all category) | Payment scheme approaches may involve prepayment and prospective payment for service, provider salaried service, the alignment of payment rates with the attainment of patient/consumer outcomes, and the removal or alteration of billing limits, such as numbers of encounters that are reimbursable. Payment may also be based on measures of treatment fidelity. Payment schemes are implementation strategies to the degree that they free the clinician’s time to provide the clinical innovation. Other strategies motivate clinicians to provide better service. | Pre-payment and prospective payment for service, provider salaried service, the alignment of payment rates with the attainment of patient/consumer outcomes, and the removal or alteration of billing limits (such as numbers of encounters that are reimbursable). These are implementation strategies to the degree that they free the clinician to provide the clinical innovation. Others motivate the clinician to provide better service | Payments scheme at organizational level.  Code if unsure about payment scheme but manuscript mentions there is one. |  |
